# STIM1-dependent Ca^2+^ signaling regulates podosome formation to facilitate cancer cell invasion

**DOI:** 10.1038/s41598-017-11273-2

**Published:** 2017-09-14

**Authors:** Yun-Wen Chen, Chieh-Shan Lai, Yih-Fung Chen, Wen-Tai Chiu, Hong-Chen Chen, Meng-Ru Shen

**Affiliations:** 1 Department of Pharmacology, College of Medicine, National Cheng Kung University, Tainan, Taiwan; 2 Graduate Institute of Natural Products, College of Pharmacy, Kaohsiung Medical University, Kaohsiung, Taiwan; 3 Department of Biomedical Engineering, National Cheng Kung University, Tainan, Taiwan; 4 Department of Obstetrics and Gynecology, National Cheng Kung University Hospital, Tainan, Taiwan; 5 Department of Life Sciences, National Chung Hsing University, Taichung, Taiwan; 6 Graduate Institute of Biomedical Sciences, National Chung Hsing University, Taichung, Taiwan; 7 Rong-Hsing Research Center for Translational Medicine, National Chung Hsing University, Taichung, Taiwan; 8 Institute of Biochemistry and Molecular Biology, National Yang Ming University, Taipei, Taiwan

**Keywords:** Cancer microenvironment, Calcium signalling

## Abstract

The clinical significance of STIM proteins and Orai Ca^2+^ channels in tumor progression has been demonstrated in different types of cancers. Podosomes are dynamic actin-rich cellular protrusions that facilitate cancer cell invasiveness by degrading extracellular matrix. Whether STIM1-dependent Ca^2+^ signaling facilitates cancer cell invasion through affecting podosome formation remains unclear. Here we show that the invasive fronts of cancer tissues overexpress STIM1, accompanied by active store-operated Ca^2+^ entry (SOCE). Interfering SOCE activity by SOCE inhibitors and STIM1 or Orai1 knockdown remarkably affects podosome rosettes formation. Mechanistically, STIM1-silencing significantly alters the podosome rosettes dynamics, shortens the maintenance phase of podosome rosettes and reduces cell invasiveness. The subsequently transient expression of STIM1 cDNA in STIM1-null (STIM1^−/−^) mouse embryo fibroblasts rescues the suppression of podosome formation, suggesting that STIM1-mediated SOCE activation directly regulates podosome formation. This study uncovers SOCE-mediated Ca^2+^ microdomain that is the molecular basis for Ca^2+^ sensitivity controlling podosome formation.

## Introduction

Tumor cell migration and invasion are essential steps for cancer metastasis via disrupting the basement membrane to disseminate cancer cells from original site to distant secondary sites^1^. Podosomes are F-actin-enriched membrane protrusions at the ventral cell surface that promote invasive motility of several types of normal cells, including macrophages^2, 3^, dendritic cells^4^, vascular smooth muscle cells^5^, osteoclasts^6^, and endothelial cells^7, 8^. Many highly invasive cancer cells display the dynamic actin-rich structures similar to podosomes, named invadopodia^9^, which are associated with degradation of the extracellular matrix (ECM)^10^. Podosome rosettes are dynamic structures that have lifespans ranging from minutes to hours^11^. Each podosome rosette consists of an actin core surrounded by integrins and integrin-associated proteins^12^. The assembled podosomes recruit matrix metalloproteinases (MMPs) and facilitate focal degradation of ECM and invasion^13^. Podosome dots can undergo self-organization into podosome rosettes, which are much potent than podosome dots in promoting matrix degradation^11^.

Modulation of cytosolic Ca^2+^ levels provides versatile and dynamic signaling involved in multiple cellular functions, such as proliferation, migration, gene regulation, and apoptosis^14^. Store-operated Ca^2+^ entry (SOCE) is a major Ca^2+^ entry pathway in non-excitable cells, which involves several steps for activation, including (i) stimulation of G proteins or protein tyrosine kinases activates phospholipase C (PLC), which hydrolyzes phosphatidylinositol bisphosphate to release the second messenger inositol-1, 4, 5-trisphosphate (IP3); (ii) binding of IP3 to IP3 receptor in the endoplasmic reticulum (ER) membrane causes a rapid and transient Ca^2+^ release from ER lumen; (iii) the decrease of ER luminal Ca^2+^ activates SOCE in the plasma membrane, leading to a sustained influx of extracellular Ca^2+^ across the plasma membrane^15, 16^. Two families of proteins, STIM (stromal-interaction molecule) and Orai, are the molecular identities responsible for SOCE activation^17, 18^. STIM proteins function as ER Ca^2+^ sensors that detect ER store depletion. Once ER Ca^2+^ is depleted, STIM proteins aggregate into multiple puncta that translocate to the close proximity of plasma membranes. Orai, an essential pore-forming component of SOCE, translocates to the same STIM-containing structures during ER Ca^2+^ depletion and opens to mediate Ca^2+^ entry.

The functional significance of SOCE in regulating cancer cell migration, invasion, and metastasis has been addressed by the studies on breast and cervical cancer cells^19–21^. Inhibition of STIM1-mediated Ca^2+^ entry, by a pharmacological inhibitor SKF96365 or by siRNA-mediated silencing of STIM1 or Orai1, caused the impairment of the focal adhesion turnover and invasive migrations of breast cancer cells^21^. Yang *et al*. also showed that blockade of SOCE inhibits hepatocarcinoma cell migration and invasion by decreasing focal adhesion turnover^22^. The STIM1/Orai1-dependent SOCE is also implicated in tumor cell migration of nasopharyngeal carcinoma and human glioblastoma^23, 24^. Our previous study showed that STIM1/Orai1-dependent SOCE enhances the cell migration of cervical cancer cells through activating the Ca^2+^-dependent protease calpain and tyrosine kinase Pyk2^19^. A recent study demonstrated that STIM1- and Orai1-mediated SOCE promotes melanoma invasion and ECM degradation by increasing invadopodia formation and activity^25^.

Translocation of the Ca^2+^-permeable TRPV2 channel may involve in the assembly of the podosome in mouse macrophages^26^. Microglial podosomes are enriched in the CRAC/Orai1 channel and are closely associated with STIM1^27^. However, the correlation between STIM1-mediated Ca^2+^ signaling and podosome rosettes formation remains elusive. Here we demonstrated that STIM1-mediated Ca^2+^ signaling is required for the formation of podosome rosettes. Blockade of STIM1-mediated Ca^2+^ signaling, by a pharmacological inhibitor SKF96365 or by siRNA-mediated silencing of STIM1, not only reduces the maintenance of podosome rosette, but also impairs the fate of podosome formation. Additionally, our results showed that the inhibition of STIM1-mediated Ca^2+^ signaling decreases podosome-mediated matrix degradation and cell invasion, supporting a role of STIM1-mediated Ca^2+^ signaling in malignant tumor progression. We propose that STIM1-mediated Ca^2+^ signaling may regulate podosome rosettes dynamics through the effect on the maintenance phase of podosome rosettes.

## Results

### Cancer tissues overexpress STIM1 and store-operated Ca^2+^ entry (SOCE)

**Figure 1:**
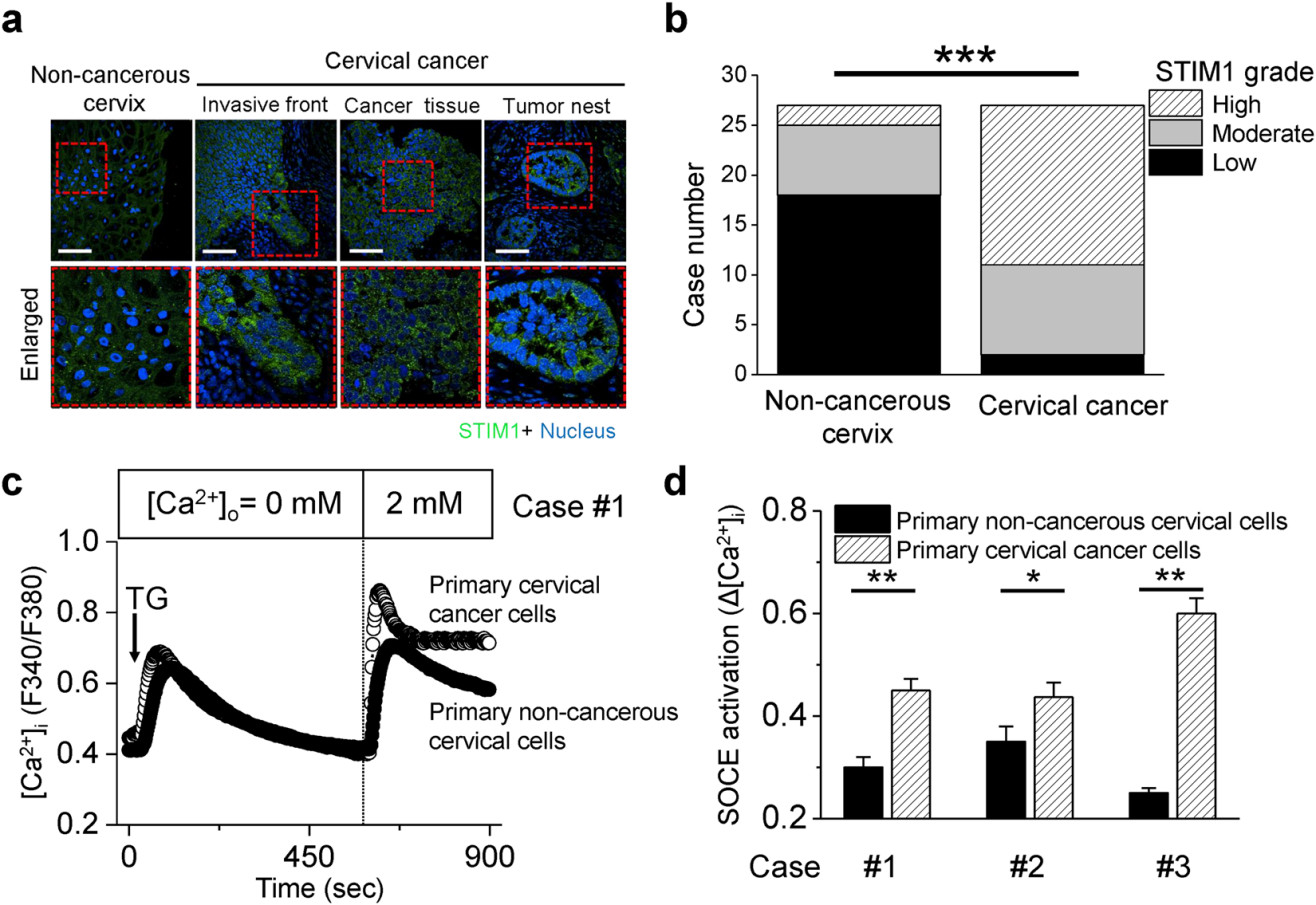
Cancer tissues overexpress STIM1 and SOCE. (**a**) Representative images of immunofluorescent staining for STIM1 expression in surgical specimens of cervical cancer. Nuclei were stained with Hoechst 33258 (blue). Scale bars, 50 μm. (**b**) The stacked column chart illustrating the intensity grade for STIM1 expression in surgical specimens of cervical cancer (n = 27). ***P < 0.001 by Chi-square test. (**c**) Representative intracellular Ca^2+^ ([Ca^2+^]_i_) measurement in primary tissue cultures derived from paired cancer and adjacent non-cancerous tissues (Case #1). Each trace is the mean [Ca^2+^]_i_ measurement of at least 30 cells. The SOCE amplitude indicates the rise of [Ca^2+^]_i_ in the replenishment of extracellular calcium ([Ca^2+^]_o_) from 0 to 2 mM. Arrow, adding 2 μM thapsigargin (TG). (**d**) Quantitative analyses of SOCE at the primary tissue cultures from 3 cervical patients with paired cancer and adjacent non-cancerous tissues. Each value represents mean ± S.E.M. of at least 30 cells. *P < 0.05, **P < 0.01, by unpaired t test.

To study the clinical relevance of STIM1 in tumor invasion, the expression patterns of STIM1 were examined in the surgical specimens of cervical cancer (Fig. 1a). Interestingly, STIM1 was abundant at the invasive front of cervical carcinoma, an area where squamous cell carcinoma just broke through the basal layers of squamous epithelia (Fig. 1a). Tumor nest was formed when cervical carcinoma invaded deeply into stromal tissues, where STIM1 was also abundant (Fig. 1a). To quantify STIM1 expression, we graded the surgical specimens by the distribution and intensity of immunofluorescent staining (Fig. 1b). The majority of these enrolled cases (18/27 = 67%) showed low grade of STIM1 expression in non-cancerous cervix. On the other hand, 92% of cases showed intermediate or high grade of STIM1 expression in the paired tumor tissues (P < 0.001, compared to the intensity grades for STIM1 expression in non-cancerous cervix).

A key question that remained unanswered is “if there is a differential SOCE activation between cancer and non-cancerous cells?” We previously compared the SOCE activation between cervical cancer cell lines and primary tissue cultures from normal subjects^28^. The results implied that cervical cancer cell lines (SiHa and CaSki) presented a more active SOCE than that of normal cervical epithelial cells derived from the normal subject. To avoid bias from the clonal effect of cell lines, here we did the primary tissue cultures from 3 cervical patients with paired cancer and adjacent non-cancerous tissues. Primary cells were cultured at the same condition for 2 days before experiments. As shown in Fig. 1c, thapsigargin-induced Ca^2+^ release from ER store was not significantly different between primary non-cancerous cervical cells and primary cervical cancer cells. Both primary non-cancerous cervical cells and primary cervical cancer cells displayed SOCE. However, the SOCE of primary cervical cancer cells was more active (Fig. 1c and d), which is consistent with the molecular evidences demonstrating overexpressed STIM1 in cancer tissues.

### STIM1 is involved in the formation of invadopodia

**Figure 2:**
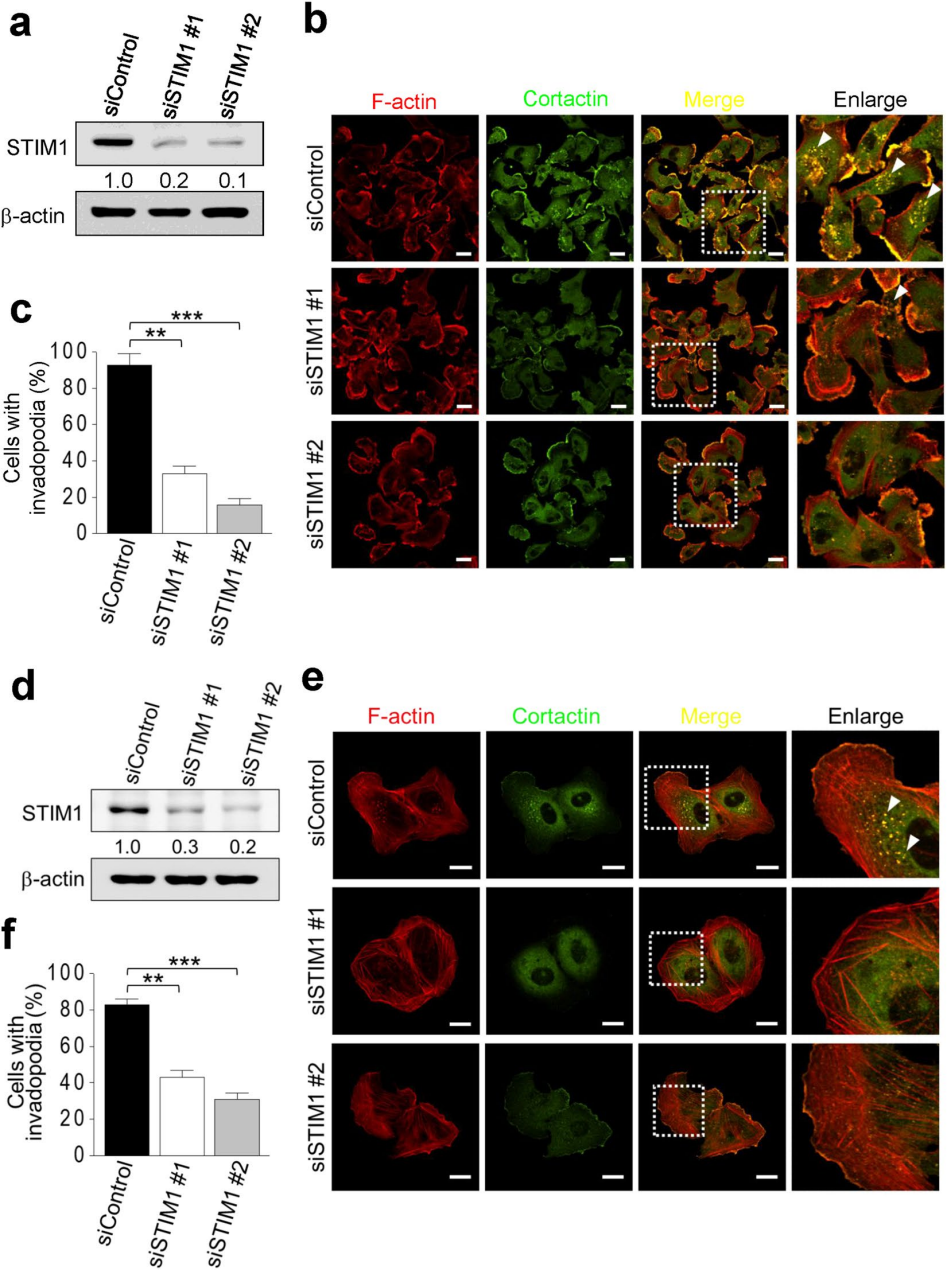
STIM1 is involved in the formation of invadopodia. Cells were transiently transfected with two STIM1-specific siRNA prior to being seeded on the gelatin-coating coverslips. (**a**) Knockdown efficiency of siControl, siSTIM1 in breast cancer MDA-MB-231 cells. Cropped blots have been presented. Full length blots are presented in Supplementary Fig. S4 (**b**) Representative confocal images showing the expression of F-actin and cortactin in breast cancer MDA-MB-231 cells. F-actin (red), cortactin (green), Scale bar, 20 μm. *Arrow*, invadopodia. (**c**) Quantitative analyses of cells with invadopodia at siControl and siSTIM1 groups in breast cancer MDA-MB-231 cells. Values represent mean ± S.E.M. from three independent experiments. **P < 0.01, ***P < 0.001, compared with control groups. (**d**) Knockdown efficiency of siControl, siSTIM1 in osteosarcoma U2OS cells. Cropped blots have been presented. Full length blots are presented in Supplementary Fig. S5 (**e**) Representative confocal images showing the expression of F-actin and cortactin in osteosarcoma U2OS cells. F-actin (red), cortactin (green), Scale bar, 20 μm. *Arrow*, invadopodia. (**f**) Quantitative analyses of cells with invadopodia at siControl and siSTIM1 groups in osteosarcoma U2OS cells. Values represent mean ± S.E.M. from three independent experiments. **P < 0.01, ***P < 0.001, compared with control groups.

Invadopodia are the dynamic F-actin-rich membrane protrusions presenting in the different types of cancer cells to promote cancer cell invasion and metastasis. We studied the role of STIM1 in the formation of invadopodia in cancer cells (Fig. 2). F-actin dots with co-localization of cortactin were considered to be an indicator for invadopodia. Invadopodia were detected in 90~100% of breast cancer MDA-MB-231 cells (Fig. 2b and c) and 80~90% of osteosarcoma U2OS cells (Fig. 2e and f), whereas suppressed STIM1 expression by two STIM1-specific siRNAs (Fig. 2a and b) significantly decreased the number of invadopodia per cell in these two cancer cell lines (Fig. 2c and f). These results suggest the association between the expression level of STIM1 and the formation of invadopodia.

### SOCE activation is required for the formation of podosome rosettes

**Figure 3:**
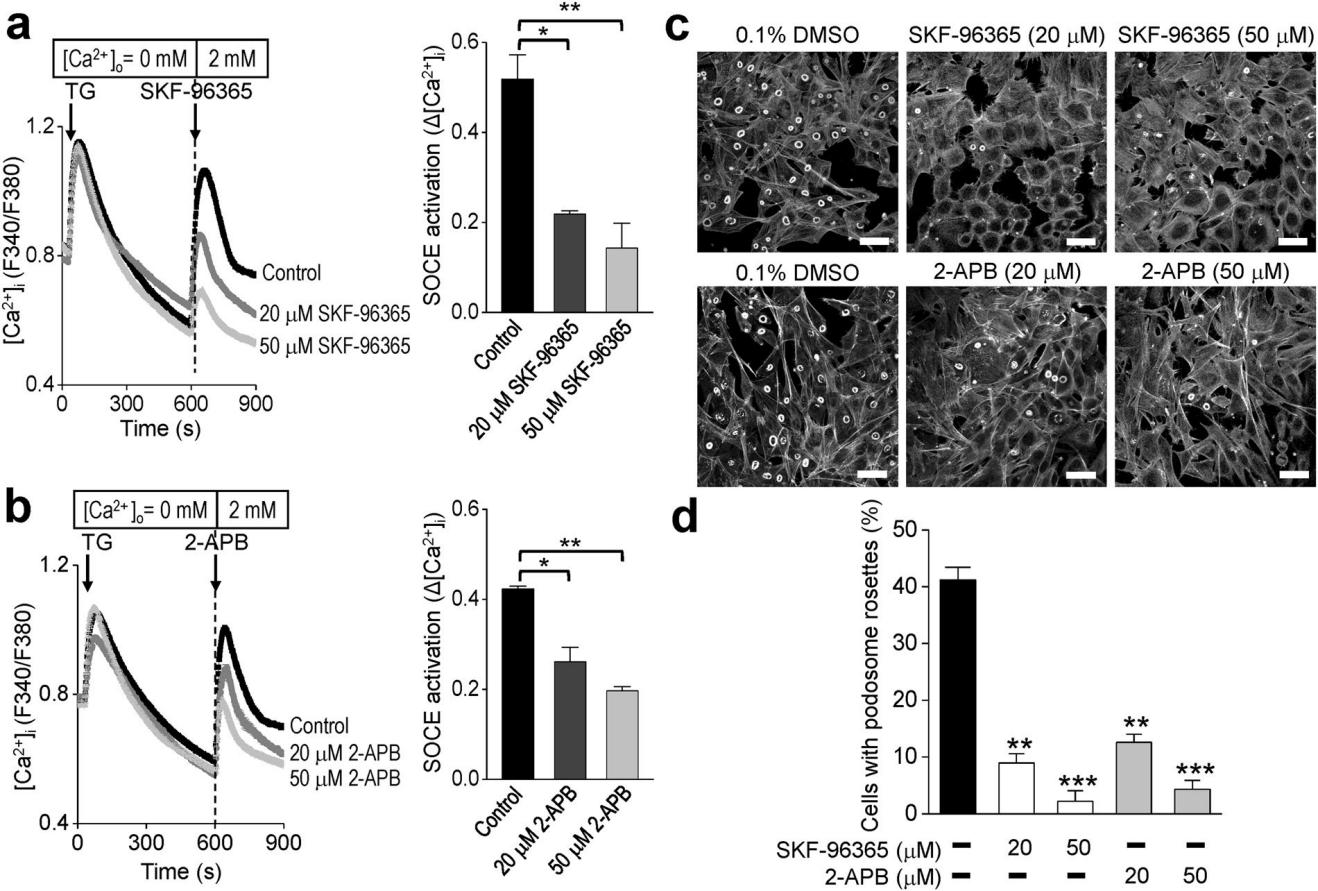
Attenuation of SOCE activity decreases the podosome rosette formation in v-Src-transformed MEFs. (**a**) *Left*, Representative intracellular Ca^2+^ ([Ca^2+^]_i_) measurement in v-Src-transformed MEFs. Each trace is the mean [Ca^2+^]_i_ measurement of at least 100 cells. The SOCE amplitude indicates the rise of [Ca^2+^]_i_ in the replenishment of [Ca^2+^]_o_ from 0 to 2 mM. *First arrow*, adding 2 μM thapsigargin (TG). *Second arrow*, adding 0.1% DMSO or SKF-96365 (20 or 50 μM). *Right*, Quantitative analyses of SOCE activity. Each value represents mean ± S.E.M. of at least 100 cells. *P < 0.01 **P < 0.001 by unpaired t test. (**b**) *Left,* representative intracellular Ca^2+^ ([Ca^2+^]_i_) measurement in v-Src-transformed MEFs. Each trace is the mean [Ca^2+^]_i_ measurement of at least 100 cells. The SOCE amplitude indicates the rise of [Ca^2+^]_i_ in the replenishment of [Ca^2+^]_o_ from 0 to 2 mM. *First arrow*, adding 2 μM thapsigargin (TG). *Second arrow*, adding 0.1% DMSO or 2-APB (20 or 50 μM). *Right*, quantitative analyses of SOCE activity. Each value represents mean ± S.E.M. of at least 100 cells. *P < 0.01 **P < 0.001 by unpaired t test. (**c**) The v-Src-transformed MEFs were pre-incubated with 0.1% DMSO or SKF-96365 (20 and 50 μM) and 2-APB (20 and 50 μM) for 24 hours. The cells were stained for F-actin. Scale bar, 30 μm. (**d**) Quantitative analyses of the cells with podosome rosettes. Values represent mean ± S.E.M. from at least 200 individual cells. **P < 0.01, ***P < 0.001, compared with control groups.

We subsequently employed a different approach to verify the important role of SOCE in cancer cell invasion. Podosomes are unique and dynamic structures located at the ventral side of invasive cells to facilitate the ECM degradation. With the cell model of v-Src-transformed mouse embryonic fibroblast (MEF) cells, we investigated the important role of SOCE in the formation of podosome rosettes. As shown in Fig. 3a and b, two SOCE inhibitors, SKF-96365 and 2-APB, blocked the activation of SOCE in a dose-dependent manner. Podosome rosettes were identified by staining with phalloidin, which binds F-actin and therefore visualizes the structures. Podosome rosettes were detected in 40%~50% of v-Src-transformed MEF cells (Fig. 3c). By contrast, only 8.95%, 2.21%, 12.57% and 4.30% of podosome rosettes displayed in v-Src-transformed MEF cells for 20 μM SKF-96365, 50 μM SKF-96365, 20 μM 2-APB, and 50 μM 2-APB groups, respectively (Fig. 3c,d). These results imply that SOCE activity is necessary for the formation of podosome rosettes in v-Src-transformed MEF cells.

### STIM1-dependent Ca^2+^ signaling is essential for controlling podosome rosettes formation

**Figure 4:**
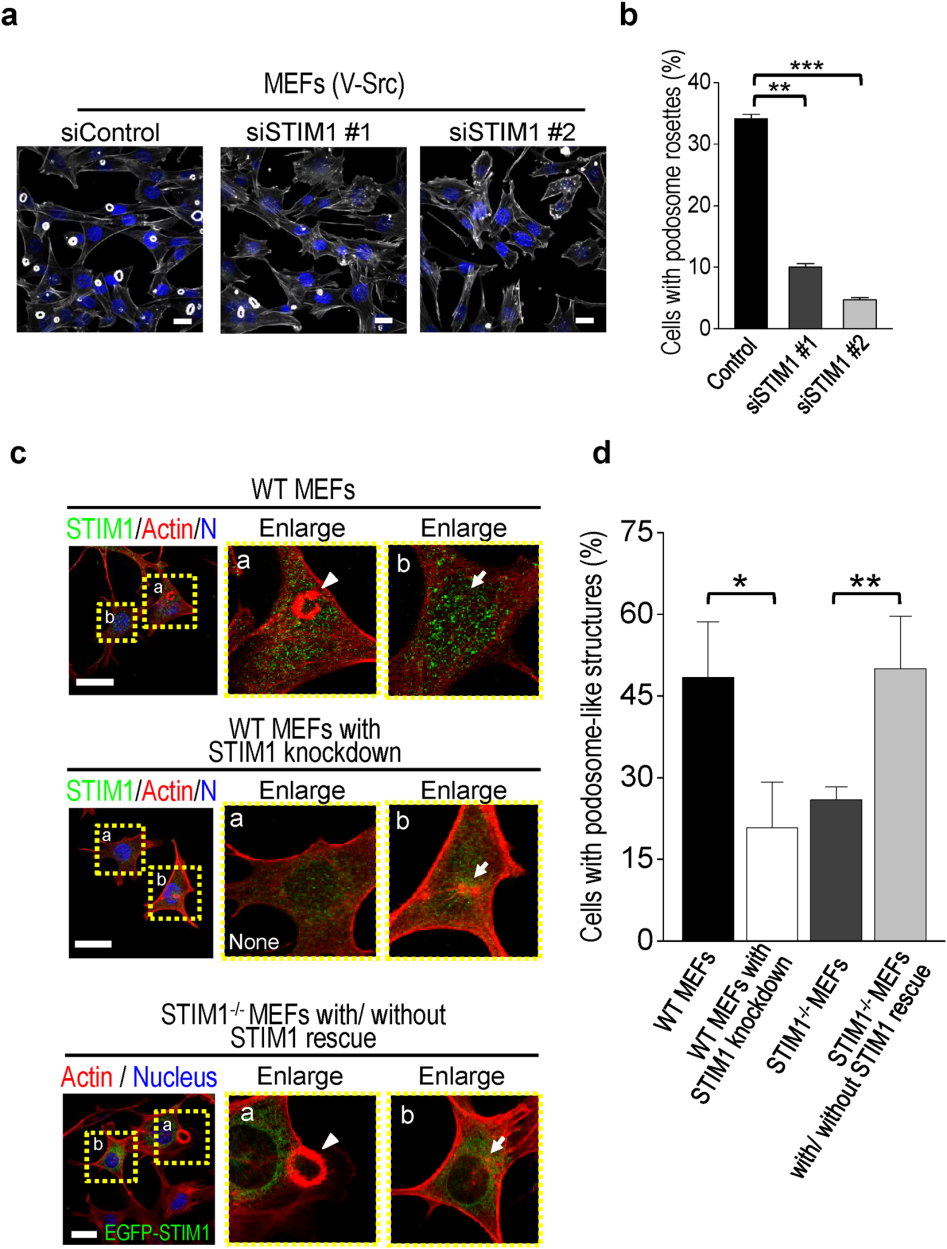
Podosome rosette formation depends on the STIM1-dependent Ca^2+^ signaling. (**a**) Representative confocal images showing the expression of F-actin. Scale bar, 20 μm. (**b**) Quantitative analyses of the cells with podosome rosettes. Silencing of STIM1 decreases the percentage of podosome rosette formation. Values represent mean ± S.E.M. from at least 200 individual cells. **P < 0.01, ***P < 0.001, compared with control groups. (**c**) Representative confocal images showing the expression of F-actin and STIM1. MEFs lacking STIM1 were re-transfected with EGFP-STIM1 plasmids. Nuclei, Hoechst 33258 (blue), F-actin (red), STIM1 (green). (**d**) Quantitative analyses of cells with podosome-like structures in wild-type MEFs, wild-type MEFs with STIM1 Knockdown, and MEF lacking STIM1 with or without STIM1 rescue. Each value represents mean ± S.E.M. from at least 30 different cells. *P < 0.01.

**Figure 5:**
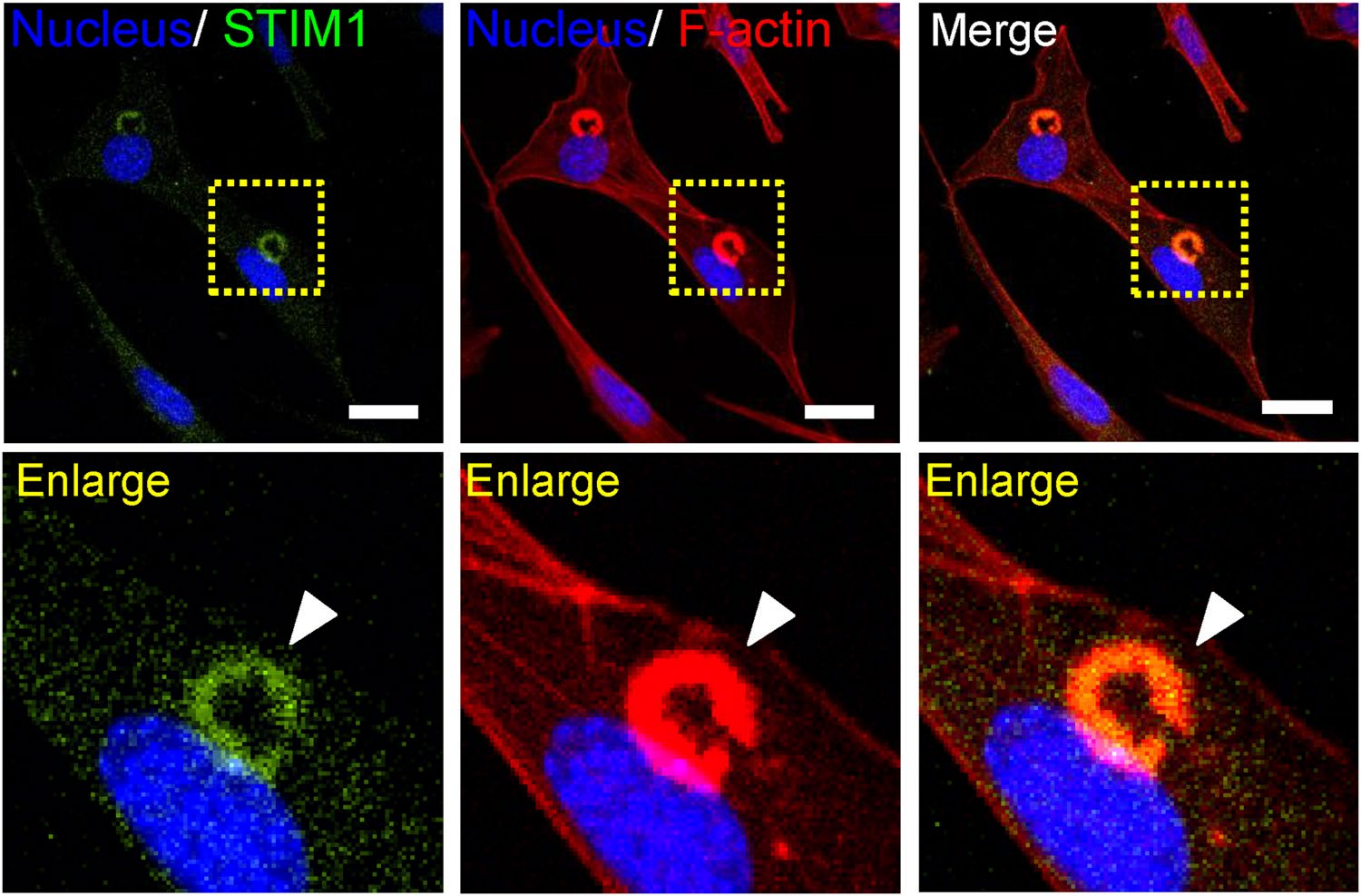
STIM1 colocalizes with podosome rosettes. Cells were grown on glass coverslips coated with 10 µg/ml fibronectin for 24 hours. Cells were fixed and then stained with STIM1 antibody labeling with AlexaFluor 488 (green), Phalloidin (red) for F-actin and Hoechst 33258 (blue) for nucleus. The images were captured by confocal microscope (Olympus, FV-1000). Scale bar, 20 μm. *Lower*, images representing the enlargement of the areas indicated by rectangles in whole-cell images (*upper*). Arrow indicated the presence of STIM1 (green), F-actin (red), and the colocalization between STIM1 and F-actin.

STIM1 and Orai1 are two key components of SOCE. We first examined whether STIM1 and Orai1 are involved in the regulation of SOCE during podosome rosette formation. STIM1 knockdown by different siRNA duplexes in v-Src-transformed MEF cells was accompanied by a significant decrease of SOCE activation (Supplementary Fig. S1a). Similarly, Orai1-specific siRNA also inhibited SOCE activation (Supplementary Fig. S1b). Knockdown of STIM1 significantly suppressed the formation of podosome rosette in v-Src-transformed MEF cells (Fig. 4a). Similarly, Orai1-specific siRNA also inhibited podosome rosette formation (Supplementary Fig. S1c). We further utilized MEF lacking STIM1 (STIM1^−/−^) to study the important role of STIM1-mediated SOCE in controlling podosome rosettes formation. In wild-type MEF, we defined podosome dots and rosettes altogether known as podosome-like structures. The significant decrease of podosome-like structures was noted in STIM1 knockdown groups (Fig. 4c and d). In the STIM1^−/−^ MEFs, there were 26% of podosome-like structures, similar to STIM1 knockdown in WT MEFs. The effect of STIM1 knockdown on the formation of podosome-like structures was rescued by the subsequent transient expression of STIM1 cDNA in STIM1^−/−^ MEF (Fig. 4c and d). Interestingly, the confocal microscopic analyses revealed that STIM1 was colocalized with podosome rosettes in v-Src–transformed MEFs (Fig. 5). These data suggest that STIM1-mediated Ca^2+^ signaling is necessary for podosome rosettes formation.

### Blockade of STIM1-mediated Ca^2+^ signaling alters the dynamics of podosome rosettes

**Figure 6:**
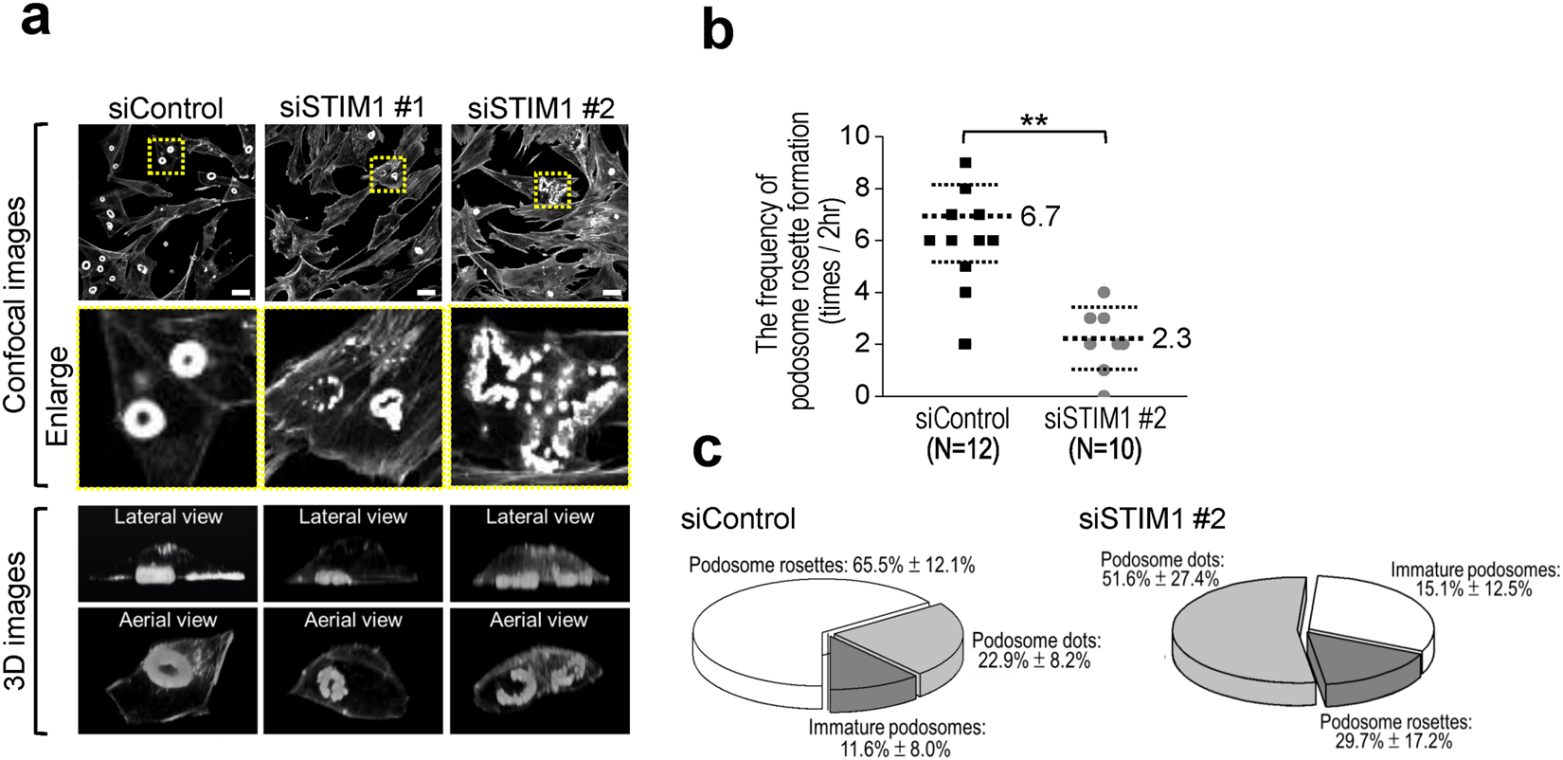
Knockdown of STIM1 disrupts the structure of podosome rosettes. (**a**) *Upper*, representative confocal images showing the expression of F-actin. Scale bar, 20 μm. Arrow, the incomplete podosomes. *Lower*, representative 3D images of podosome rosettes visualized by the Avizo software from at least 40 slices of confocal images. (**b**) v-Src-transformed MEFs stably expressing GFP-actin were recorded by time-lapse microscopy for 2 hours to examine the podosome dynamics in the presence or absence of STIM1-specific siRNA. The frequency of podosome rosette formation is decreased in the STIM1 knockdown group. The frequency of podosome rosette formation in the total counted podosome rosettes (n = 12 and 10) was determined. **P < 0.01. (**c**) v-Src-transformed MEFs stably expressing GFP-actin were recorded by time-lapse microscopy for 2 hours to examine and analyze the types of podosome in the presence or absence of STIM1-specific siRNA. STIM1-silencing changes the podosome populations. There are three major groups of podosome population in v-Src-transformed MEFs, including dots, immature podosomes, and rosettes.

During the process of podosome formation, podosomes display different shapes including dots, immature podosomes, rosettes and belts in v-Src-transformed MEFs. To examine the role of STIM1-mediated Ca^2+^ signaling in the formation of podosome rosettes, we visualized the podosome structure by employing the AVIZO software to illustrate the three-dimensional (3D) images of podosome rosettes. As shown in Fig. 6a, the structure of podosome rosettes at the ventral cell surface was dense donut-like shape in the control group. In contrast, the structure of podosome rosettes became disrupted and fragmented in the STIM1 knockdown group. To quantitatively study the role of STIM1-mediated Ca^2+^ signaling during podosome rosettes formation, we monitored podosome dynamics by time-lapse recording in living cells. Cells were recorded for 2 hours for observing and analyzing the podosome dynamics. We found that inhibition of STIM1-mediated Ca^2+^ signaling changed the dynamics in the formation of podosome rosettes (Fig. 6b). Moreover, STIM1 knockdown by siRNA changed the distribution of podosome population (Fig. 6c). As shown in Fig. 6c, the major population of podosome in control group was 65.5% with rosettes, but the main population (51.6%) was podosome with dots in the STIM1 knockdown group. Taken together, these results indicate that blocking STIM1-mediated Ca^2+^ signaling alters the podosome rosettes formation by regulating the dynamics of podosome rosettes.

### Blockade of STIM1-mediated Ca^2+^ signaling shortens the maintenance phase of podosome rosettes

**Figure 7:**
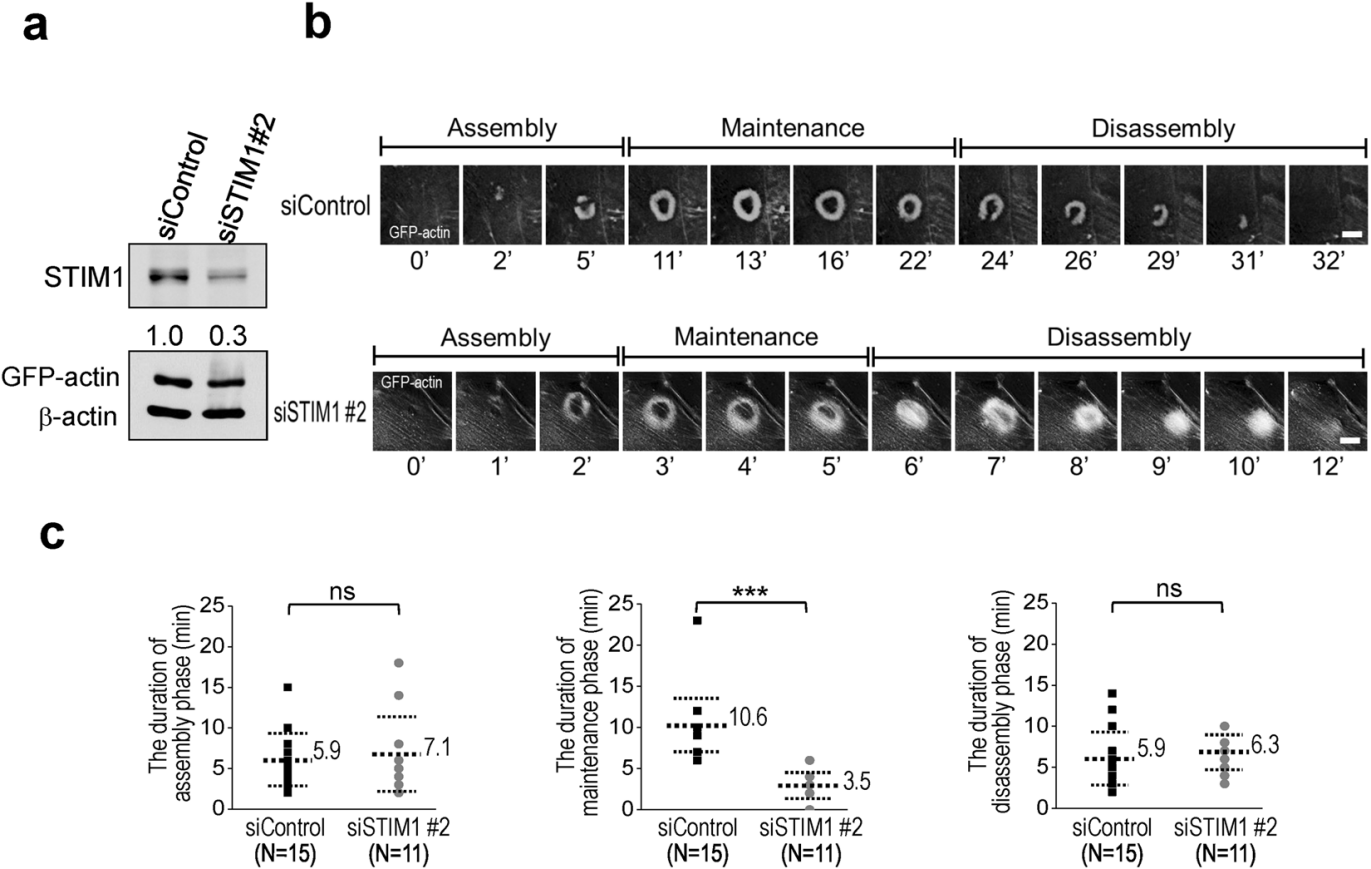
Blockade of STIM1-mediated Ca^2+^ signaling shortens the maintenance phase of podosome rosette formation. (**a**) Knockdown efficiency of siControl, siSTIM1 in v-Src-transformed MEFs. Cropped blots have been presented. Full length blots are presented in Supplementary Fig. S6 (**b**) The process of podosome rosette formation is illustrated by real-time recording. v-Src-transformed MEFs stably expressing GFP-actin were monitored by time-lapse microscopy for 2 hours to examine the podosome dynamics in the presence or absence of STIM1-specific siRNA. Representative sequential images selected at different time point are shown. Scale bar, 5 μm. (**c**) The quantitative duration of the assembly phase, maintenance phase in the total counted podosome rosettes (n = 15) was determined in the presence or absence specific STIM1 siRNA. ***P < 0.001. ns, not significant.

The duration of podosome is from minutes to hours, which varies in different cell types^12^. The formation of podosome rosette is divided into three stages: assembly, maintenance, and disassembly^11^. We examined the effect of STIM1-mediated Ca^2+^ signaling on the stages of podosome rosette formation using living-cell image recording. The series of time-lapse images revealed that the maintenance phase of podosome rosettes of the STIM1 knockdown group is shorter than that of the control group (Supplementary material Movie  1 and 2). As shown in Fig. 7b and c, there was no significant difference between control group and STIM1 knockdown group in the assembly and disassembly stages. However, STIM1 knockdown significantly shortened the duration of maintenance stage. These results indicate that inhibition of STIM1-mediated Ca^2+^ signaling decreases the formation of podosome rosettes via downregulation of the maintenance stage of podosome rosette.

### STIM1-mediated Ca^2+^ signaling is important for secreted MMPs activity, podosome-mediated matrix degradation, and cell invasion

**Figure 8:**
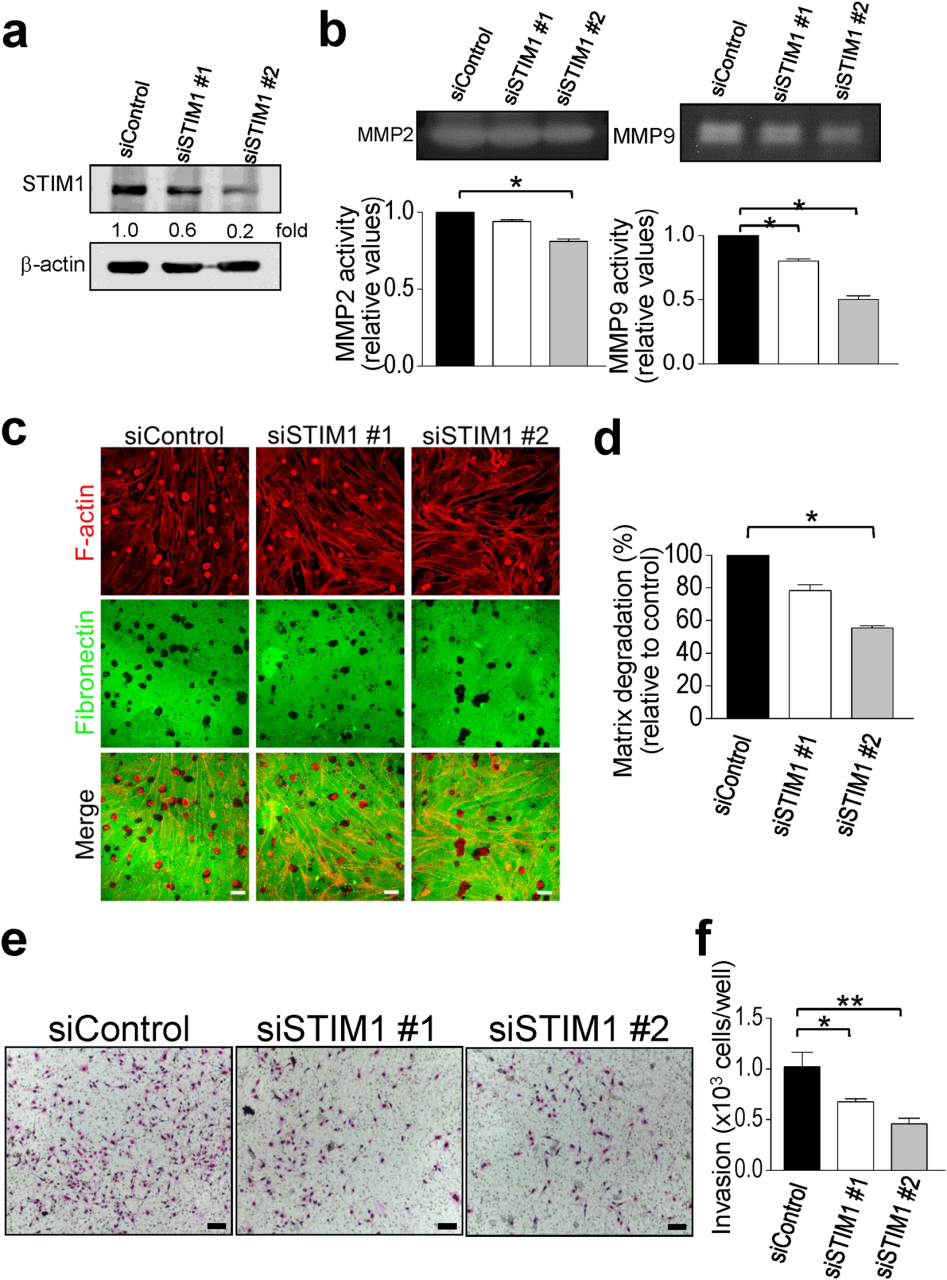
STIM1-mediated Ca^2+^ signaling relates the secreted MMPs activity, podosome-mediated matrix degradation, and cell invasion. (**a**) Knockdown efficiency of siControl, siSTIM1 in v-Src-transformed MEFs. Cropped blots have been presented. Full length blots are presented in Supplementary Fig. S7 (**b**) Representative zymograms showing activities of MMP2 and MMP9 in the conditioned medium. Equal volume (40 μL) of conditioned medium collected from 10-cm dish with equal cell number was loaded into each well. Columns represent mean ± S.E.M. from three independent experiments. *P < 0.05, compared with control group. Cropped blots have been presented. Full length blots are presented in Supplementary Fig. S8 (**c**) Cells were transient transfection with two STIM1-specific siRNA prior to being plated on AlexaFluor 488-conjugated fibronectin (FN). (**d**) Quantitative analyses of the areas in which fibronectin had been degraded were measured. Values represent mean ± S.E.M. from three independent experiments. *P < 0.05, compared with control groups. (**e**) Transwell assay with Matrigel was performed to detect the invasion activity of v-Src-transformed MEFs transfected with siControl or siSTIM1. Scale bar, 50 μm. (**f**) Quantitative analyses of the cells migrating through the Matrigel coated filter. *P < 0.01, compared to the control groups. Values represent mean ± S.E.M. from three independent experiments. *P < 0.05, **P < 0.01, compared with control groups.

Tumor invasion is a complicated process that requires multiple cellular functions, such as invadopodia formation, expression of MMPs, and cell motility. One of the major functions of podosome rosettes is to degrade ECM that contributes to cell motility and invasion^13^. MMP2, MMP9, and MT1-MMP play important roles in podosome-mediated matrix degradation. To determine the role of STIM1-mediated Ca^2+^ signaling in MMP activity and matrix degradation of podosome rosettes, we used gelatin zymography to detect the activity of major secreted MMPs. MMP2 and MMP9 activities significantly decreased in the STIM1 knockdown group, compared with control group (Fig. 8b). Moreover, the control group showed the colocalization between podosome rosettes and the sites of matrix degradation. By contrast, the degradation area of fibronectin was less in the STIM1 knockdown group (Fig. 8c). The quantitative results showed that inhibition of STIM1-mediated Ca^2+^ signaling was correlated with decreases in the areas of degradation (Fig. 8d). Together, these results indicated that STIM1-mediated Ca^2+^ signaling not only regulates the podosome rosette formation, but also meditates its degradation ability. We further examined whether the downregulation of matrix degradation contributed to decreasing cell invasion. Matrigel-based Transwell invasion assays were performed to investigate the effects of STIM1-mediated Ca^2+^ signaling on cell invasion. The data showed that blockade of STIM1-mediated Ca^2+^ signaling significantly inhibited the cell invasion in a dose-dependent manner (Fig. 8e and f). Taken together, those results indicate the crucial role for STIM1-mediated Ca^2+^ signaling in podosome rosette-mediated cell invasion.

## Discussion

This study highlights that STIM1-mediated Ca^2+^ signaling is necessary for the formation of podosome rosette in v-Src-transformed MEFs. This conclusion is supported by the following evidences. (a) The siRNA-mediated knockdown of STIM1 or Orai1 expression or the pharmacologic inhibition of SOCE activity significantly inhibited podosome rosette formation. (b) Blockade of STIM1-mediated Ca^2+^ signaling disrupted the structure of podosome rosettes and shortened the maintenance phase of podosome rosette formation. (c) Results from MEF cells lacking STIM1 confirmed that the regulation of podosome rosette formation required STIM1-dependent SOCE activation. (d) Blockade of STIM1-mediated Ca^2+^ signaling inhibited cancer cell migration and invasion.

The importance of Ca^2+^ signaling in the regulation of cell migration and cell-substrate adhesions has been studied in different cell models. Invadopodia, also called podosomes in non-malignant cells, are actin-rich membrane protrusions mediating focal ECM degradation and invasive motility in highly invasive cancer cells. However, the exact molecular identities of Ca^2+^ transport proteins and the regulatory mechanisms of the Ca^2+^ signaling pathways involved in podosome formation have remained elusive. A recent work by Sun *et al*. showed that STIM1- and Orai1-mediated SOCE promotes melanoma invasion and ECM degradation by increasing invadopodia formation and activity^25^. Using pharmacological inhibitors, Sun *et al*. demonstrated that SOCE is required for invadopodia maturation and mediating focal ECM degradation^25^. Here we focused on the novel role of STIM1-mediated Ca^2+^ signaling in regulating the structural integrity and dynamics of podosome rosettes. The 3D images of podosome rosettes revealed that siRNA-mediated knockdown of STIM1 causes the structural disruption and fragmentation of podosome rosettes. Moreover, results from living-cell image recording of podosome dynamics showed that STIM1 silencing significantly decreased the duration of maintenance phase, suggesting that STIM1-mediated Ca^2+^ signaling affects the certain phase in podosome rosette dynamics.

Podosomes can self-organize into large rosette-like structures in some types of cells. The podosome dots did not naturally assemble into podosome rosettes in the absence of FAK^11^. The Rho-ROCK mediated signaling pathway can promote actomyosin-based cell contraction and contribute to podosome disassembly^29^. Our previous study proved that STIM1-dependent Ca^2+^ signaling controls cell migration by the regulation of actomyosin reorganization^30^. In this study, we demonstrated that knockdown of STIM1 shortened the maintenance phase of podosome rosettes, probably due to STIM1-mediated actomyosin distribution. Our results showed that the distribution of phospho-myosin light chain II was altered by blocking the STIM1-mediated Ca^2+^ signaling (Fig. S3). Of note, the total expression of phosphor-myosin light chain II remained unchanged. These results imply that the actomyosin plays an important role in the podosome rosette formation.

The importance of Ca^2+^ signaling in the regulation of podosome formation has been studied in different cell models, but the way in which it controls podosome formation yet remains elusive. Here we show that STIM1-Orai1 pathway of SOCE plays an important role in regulating podosome formation. The depletion of STIM1 by siRNA showed a significant decrease of podosome-like structures in STIM1 knockdown groups. More importantly, the STIM1 rescue study in STIM1^−/−^ MEF confirmed that STIM1-mediated SOCE activation directly mediates podosome formation. These results suggest that STIM1-mediated SOCE activation is the major determinant of the Ca^2+^ sensitivity regulating podosome formation.

In conclusion, we demonstrated that STIM1-mediated Ca^2+^ signaling is important for the formation of podosome rosettes formation and maintenance of podosome rosettes. Moreover, blockade of STIM1-mediated Ca^2+^ signaling inhibits the capability of cell invasion.

## Materials and Methods

### Surgical specimens

For primary tissue cultures, we enrolled 3 cases with pair tissues of cervical carcinoma and adjacent non-cancerous epithelia. Another 27 cases of paraffin blocks containing tissues of cervical carcinoma and adjacent non-cancerous epithelia were for immunofluorescent staining. All these cases are patients with early-stage (International Federation of Gynecology and Obstetrics staging Ib) cervical cancer who underwent surgery at National Cheng Kung University Hospital, Taiwan. The collection of surgical specimens was approved by the institutional review board of National Cheng Kung University Hospital. All experimental protocols and procedures were performed in accordance with relevant guidelines and regulation, and all participants provided written informed consent.

### Cell cultures, transfection, RNA interference

v-Src-transformed MEFs and GFP-actin v-Src-transformed MEFs were grown in high-glucose DMEM (GIBCO) with 10% fetal bovine serum (GIBCO), 100 μM non-essential amino acids, 1 mM sodium pyruvate, and 55 μM 2-mercaptoethanol and cultured at 37 °C in a humidified atmosphere of 5% CO2 and 95% air. GFP-actin v-Src-transformed MEFs were selected using Zeocin (Invitrogen). Osteosarcoma cell line U2OS was grown in low-glucose DMEM (GIBCO) with 10% fetal bovine serum (GIBCO). Breast cancer MDA-MB-231 cells were cultured in RPMI-1640 (GIBCO) with 10% fetal bovine serum (GIBCO). Two independent pairs of different siRNAs (Sigma) and a siRNA pool of three duplexes (Santa Cruz Biotechnology) targeting STIM1 or Orai1 were used in this study.

### Antibodies, chemicals, and immunoblotting

Antibodies against STIM1 (1:1000), Myosin II (1:1000) and phospho-Ser19 Myosin light chain II (1:1000) were purchased from Cell Signaling Technology. Antibody against β-actin (clone AC-15) (1:5000) was purchased from Sigma-Aldrich. Antibodies against MMP2 (1:1000) and MMP9 (1:1000) were purchased from Millipore. Antibodies against MT1-MMP (1:500) and cortactin (1:100) were purchased from Santa Cruz Biotechnology. Thapsigargin, SKF-96365, and 2-APB were from Cayman Chemical. For immunoblotting, cells were harvested with ice-cold modified radioimmune precipitation assay (RIPA) buffer containing a protease inhibitor mixture (Roche Diagnostics), 100 mM KCl, 80 mM NaF, 10 mM EGTA, 50 mM h-glycerophosphate, 10 mM p-nitrophenyl phosphate, 1 mM vanadate, 0.5% sodium deoxycholate, and 1% NP40. Protein concentrations were determined with the use of a Bio-Rad protein assay. Equal amounts of protein lysates were separated by SDS-PAGE, and then transferred to nitrocellulose blotting membranes (Pall). Immunoblots were blocked, incubated with the primary antibody, washed, and incubated with the corresponding horseradish peroxidase-conjugated secondary antibody (Jackson ImmunoResearch), and visualized by Western blotting luminol reagent (Santa Cruz Biotechnology). Bands in the immunoblots were quantified using Vision WorksLS software (UVP).

### Immunofluorescence staining, confocal microscopy, deconvolution microscopy and image analyses

To quantify STIM1 expression, we graded the surgical specimens by the distribution and intensity of immunofluorescent staining. The high grade indicates that immunofluorescent staining intensity and distribution are more than 70% of the observed area, whereas low grade indicates that the staining intensity and distribution are less than 30% of observed area. The rest of the group was graded as “intermediate”. For immunofluorescent staining, cells were seeded on glass coverslips. Cells were fixed by 4% paraformaldehyde, permeabilized by 0.1% Triton X-100 for 15 min, and blocked with commercial blocking serum (Invitrogen). These cells were incubated with primary antibody at 4 °C overnight, washed, and then incubated with AlexaFluor-conjugated secondary antibodies (Invitrogen) for 1 hour at room temperature. To detect nuclei and actin filaments, cells were stained with Hoechst 33258 and phalloidin conjugated with tetramethylrhodamine B isothiocyanate (TRITC; Sigma-Aldrich) for 1 hour at room temperature. Cells were washed and mounted, and the fluorophores were excited by laser at 405, 488, or 594 nm and detected by a scanning confocal microscope (FV-1000, Olympus). To examine the role of STIM1 in the formation of podosome rosettes, the 3D images of F-actin were reconstructed from serial confocal Z-section scanning images using AVIZO 3D imaging and analysis software (Version 6.0, Mercury computer systems).

### Time-lapse live cell recording

v-Src-transformed MEF cells that stably expressed GFP-actin were grown on glass coverslips coated with 10 µg/ml fibronectin. The cells on the microscope stage were maintained at 37 °C in a humid CO_2_ atmosphere in a micro-cultivation system with temperature and CO_2_ control devices. The cells were monitored by a deconvolution microscopy (DeltaVision Elite, GE Healthcare). Images were captured every 1 min for 2 hours and analyzed by softWoRx® software. Following our previous study on podosome dynamics^31^, three stages of podosome formation were defined. [1] Assembly phase: Dot-shaped podosomes, which are the main structures of podosomes, are allowed to assemble into podosome rosettes. [2] Maintenance phase: Once fully assembled, the podosome itself exhibits the mechanosensory characteristics and podosome rosettes remain complete ring structures. [3] Disassembly phase: The disassembly of podosome rosettes is manifested by the collapse of the F-actin ring structure toward its center before it is completely dissolved.

### Single cell [Ca^2+^]_i_ measurement

Intracellular Ca^2+^ was measured at 37 °C with the Fura-2 fluorescence ratio method on a single-cell fluorimeter. In brief, cells attached on glass-bottom dishes were loaded with 2 μM Fura-2/acetoxymethyl ester (Fura-2/AM) in serum-free culture medium at 37 °C for 30 min. Cells were then washed three times with PBS. The dish was then placed on the stage of an Olympus IX71 inverted microscope equipped with a xenon illumination system and an IMAGO CCD camera (TILL Photonics). The excitation wavelength was alternated between 340 nm (I340) and 380 nm (I380) using the Polychrome IV monochromator (TILL Photonics). The fluorescence intensity of excitation at 510 nm was monitored to calculate the intracellular Ca^2+^ levels by TILLvisION 4.0 program (Till Photonics). ER Ca^2+^ release was induced with 2 μM thapsigargin for 10 minutes in the absence of extracellular Ca^2+^, followed by the activation of SOCE with the addition of 2 mM Ca^2+^.

### Matrix degradation assay

AlexaFluor 488-conjugated fibronectin was prepared according to the manufacturer’s instructions (Invitrogen). Cells were seeded on glass coverslips coated with 20 μg/ml AlexaFluor 488-conjugated fibronectin for 24 hours. The matrix degraded areas were measured by MetaMorph® software.

### Matrigel invasion assay

24-well transwell chambers (Corning Incorporated) with a membrane in 8 μm pore size were coated with 100 μl Matrigel (0.4 mg/ml, BD Biosciences). The lower chamber was loaded with 750 μl high-glucose DMEM with 10% serum. Cells were seeded into the upper chamber in 250 μl serum-free medium. After 24 hours, Cells were fixed and permeabilized by 100% methanol. Cells were stained with Giemsa stain and counted on inverted Microscopy (Olympus IX71).

### Gelatin Zymography

Gelatinolytic activity in the culture media was examined by gelatin zymography. The supernatants (25 μg of protein per lane) were examined by SDS-PAGE with 0.2% gelatin. After electrophoresis, gels were washed twice in 2.5% Triton X-100 to remove the SDS, and incubated for at least 24 hours at 37 °C in 50 mM Tris-HCl, pH 7.5, containing 0.15 M NaCl, 10 mM CaCl2, and 0.02% NaN_3_. After 24 hours, stained gel with 0.1% Coomassie brilliant blue R250 for 10 minutes. Gels were washed with 10% acetic acid to destain. The activity of MMP-2 and MMP9 was estimated by computer-assisted densitometric scanning of the 62-kD and 92-kD proteolytic bands, respectively.

### Statistics

All values were reported as mean ± S.E.M. (standard error of the mean). Student’s pair t test, unpaired t test or Chi-square test was used for statistical analyses. Differences between values were considered significant when P < 0.05.

## Electronic supplementary material

Supplementary Information

Supplementary material Movie 2

Supplementary material Movie 1
