# Author Correction: STIM1-dependent Ca^2+^ signaling regulates podosome formation to facilitate cancer cell invasion

**DOI:** 10.1038/s41598-019-53336-6

**Published:** 2019-11-15

**Authors:** Yun-Wen Chen, Chieh-Shan Lai, Yih-Fung Chen, Wen-Tai Chiu, Hong-Chen Chen, Meng-Ru Shen

**Affiliations:** 1 Department of Pharmacology, College of Medicine, National Cheng Kung University, Tainan, Taiwan; 2 Graduate Institute of Natural Products, College of Pharmacy, Kaohsiung Medical University, Kaohsiung, Taiwan; 3 Department of Biomedical Engineering, National Cheng Kung University, Tainan, Taiwan; 4 Department of Obstetrics and Gynecology, National Cheng Kung University Hospital, Tainan, Taiwan; 5 Department of Life Sciences, National Chung Hsing University, Taichung, Taiwan; 6 Graduate Institute of Biomedical Sciences, National Chung Hsing University, Taichung, Taiwan; 7 Rong-Hsing Research Center for Translational Medicine, National Chung Hsing University, Taichung, Taiwan; 8 Institute of Biochemistry and Molecular Biology, National Yang Ming University, Taipei, Taiwan

Correction to: *Scientific Reports* 10.1038/s41598-017-11273-2, published online 14 September 2017

Chieh-Shan Lai was omitted from the author list in the original version of this Article. This has been corrected in the PDF and HTML versions of the Article, and in the accompanying Supplementary Information file.

The Author Contributions section now reads:

Y.W.C., C.S.L., Y.F.C., W.T.C., H.C.C., and M.R.S. designed research; Y.W.C., C.S.L., Y.F.C., and W.T.C. performed research; Y.W.C., C.S.L., Y.F.C., W.T.C., and M.R.S. analyzed data; Y.W.C., Y.F.C., and M.R.S. wrote the article.

