# Supplementary material for: STIM1-dependent Ca2+ signaling regulates podosome formation to facilitate cancer cell invasion
Source: Sci Rep. 2017 Sep 14;7:11523. doi: 10.1038/s41598-017-11273-2 (PMC5599537; doi:10.1038/s41598-017-11273-2)
Supplement: Supplementary file 1 — Supplementary Information [file 41598_2017_11273_MOESM1_ESM.pdf]

STIM1-dependent Ca^{2+} signaling regulates podosome formation to facilitate cancer cell invasion

Yun-Wen Chen[1], Chieh-Shan Lai[1], Yih-Fung Chen[2], Wen-Tai Chiu[3], Hong-Chen Chen[5,6,7], Meng-Ru Shen[1,4]

[1]Department of Pharmacology, College of Medicine, National Cheng Kung University, Tainan, Taiwan; [2]Graduate Institute of Natural Products, College of Pharmacy, Kaohsiung Medical University, Kaohsiung, Taiwan; [3]Department of Biomedical Engineering, National Cheng Kung University, Tainan, Taiwan; [4]Department of Obstetrics and Gynecology, College of Medicine, National Cheng Kung University, Tainan, Taiwan; [5]Department of Life Sciences, [6]Graduate Institute of Biomedical Sciences, National Chung Hsing University, Taichung , Taiwan; [7]Rong-Hsing Research Center for Translational Medicine, National Chung Hsing University, Taichung , Taiwan

Supplementary Information

Supplementary Figure S1. STIM and Orai1 underlying SOCE involve in the regulation of podosome rosette formation in v-Src-transformed MEFs.

Supplementary Figure S2. (A) Knockdown efficiency of siControl, siSTIM1 in wild-type MEFs. (B) Western blot analysis of expression pattern of STIM1 in MEF lacking STIM1 ($STIM1^{-/-}$ MEF).

Supplementary Figure S3. Blockade of STIM1-mediated Ca^{2+} signaling changes distribution of actomysin in podosome rosettes.

Supplementary Figure S4. Full length images of the cropped blots presented in main Figure 2a

Supplementary Figure S5. Full length images of the cropped blots presented in main Figure 2d.

Supplementary Figure S6. Full length images of the cropped blots presented in main Figure 7a.

Supplementary Figure S7. Full length images of the cropped blots presented in main Figure 8a.

Supplementary Figure S8. Full length images of the cropped blots presented in main Figure 8b.

Supplementary Figure S9. Full length images of the cropped blots presented in main Figure S1a.

Supplementary Figure S10. Full length images of the cropped blots presented in main Figure S1b.

Supplementary Figure S11. Full length images of the cropped blots presented in main Figure S2a.

Supplementary Figure S12. Full length images of the cropped blots presented in main Figure S2b.

Supplementary Figure S13. Full length images of the cropped blots presented in main Figure S3a.

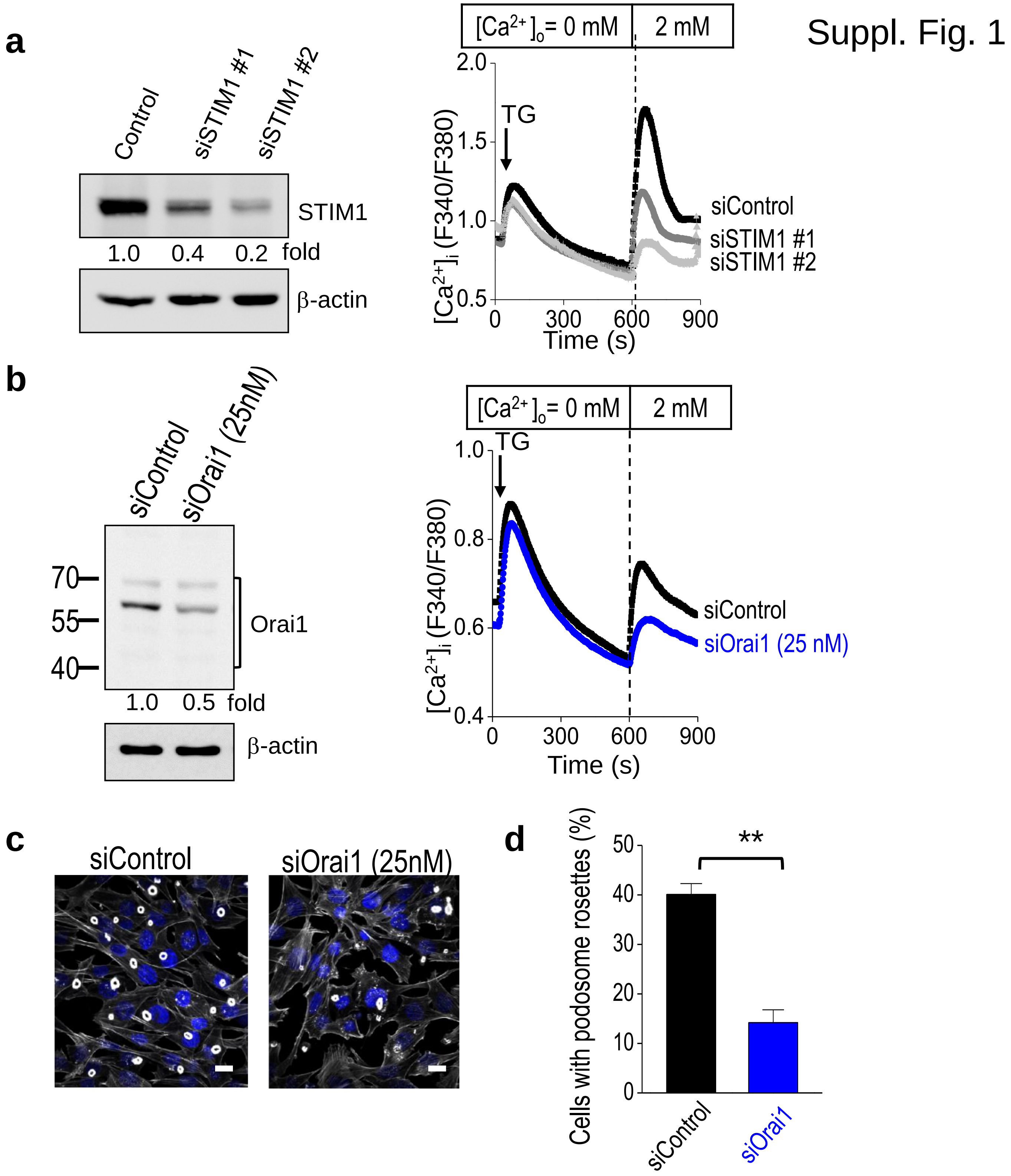

Supplementary Figure S1. STIM and Orai1 underlying SOCE involve in the regulation of podosome rosette formation in v-Src-transformed MEFs. ***(a)*** *Left*, knockdown efficiency of siControl, siSTIM1 in v-Src-transformed MEFs. Cropped blots have been presented. Full length blots are presented in Supplementary Fig. S9. *Right*, Representative intracellular Ca^{2+} ($[Ca^{2+}]i$) measurement in v-Src-transformed MEFs. Each trace is the mean $[Ca^{2+}]i$ measurement of at least 100 cells. The SOCE amplitude indicates the rise $[Ca^{2+}]i$ in the replenishment of $[Ca^{2+}]o$ from 0 to 2 mM. arrow, adding 2 μM thapsigargin (TG). ***(b)*** Left, knockdown efficiency of siControl, siOrai1 in v-Src-transformed MEFs. Cropped blots have been presented. Full length blots are presented in Supplementary Fig. S10. Right, Representative intracellular Ca^{2+} ($[Ca^{2+}]i$) measurement in v-Src-transformed MEFs. Each trace is the mean $[Ca^{2+}]i$ measurement of at least 100 cells. The SOCE amplitude indicates the rise of $[Ca^{2+}]i$ in the replenishment of $[Ca^{2+}]o$ from 0 to 2 mM. arrow, adding 2 μM thapsigargin (TG). ***(c)*** Representative confocal images showing the expression of F-actin. Scale bar, 20 μm. ***(d)*** Quantitative analyses of the cells with podosome rosettes. Silencing of Orai1 decreases the percentage of podosome rosette formation. Values represent mean ± S.E.M from at least 200 individual cells. **P<0.01, ***P<0.001, compared with control groups.

Suppl Fig.2

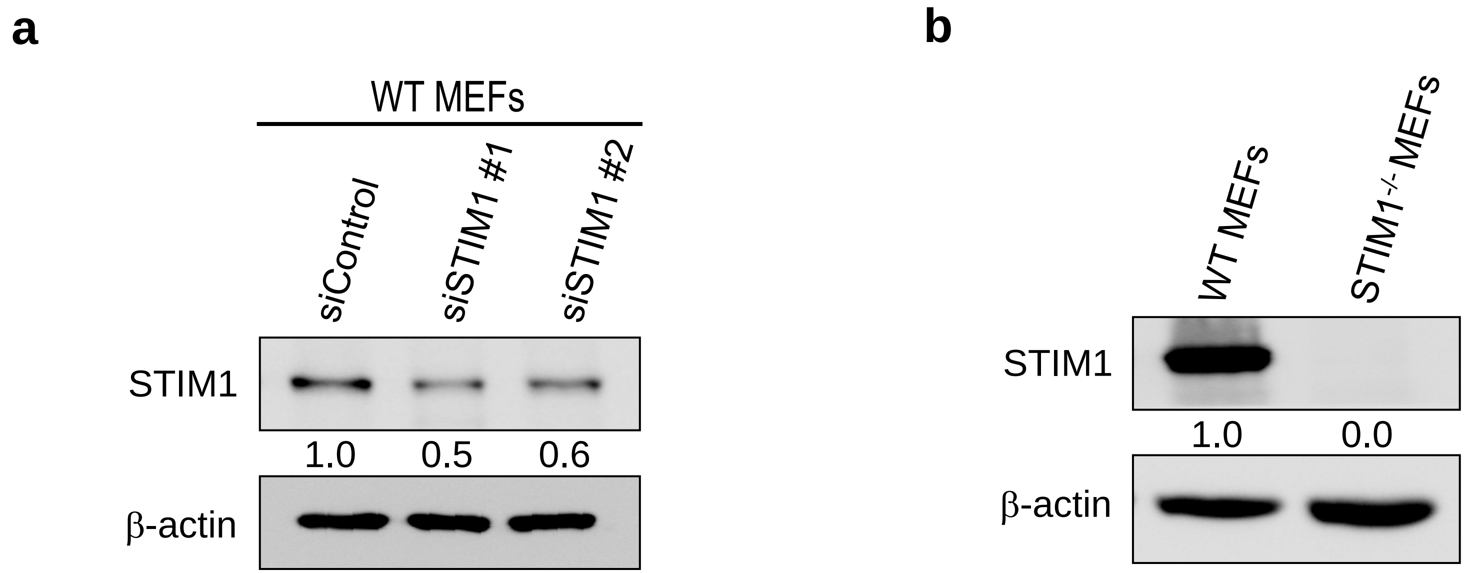

Supplementary Figure S2. ***(a)*** Knockdown efficiency of siControl, siSTIM1 in wild-type MEFs. Cropped blots have been presented. Full length blots are presented in Supplementary Fig. S11. ***(b)*** Western blot analysis of expression pattern of STIM1 in MEF lacking STIM1 ($STIM1^{-/-}$ MEF). Cropped blots have been presented. Full length blots are presented in Supplementary Fig. S12.

Suppl. Fig 3

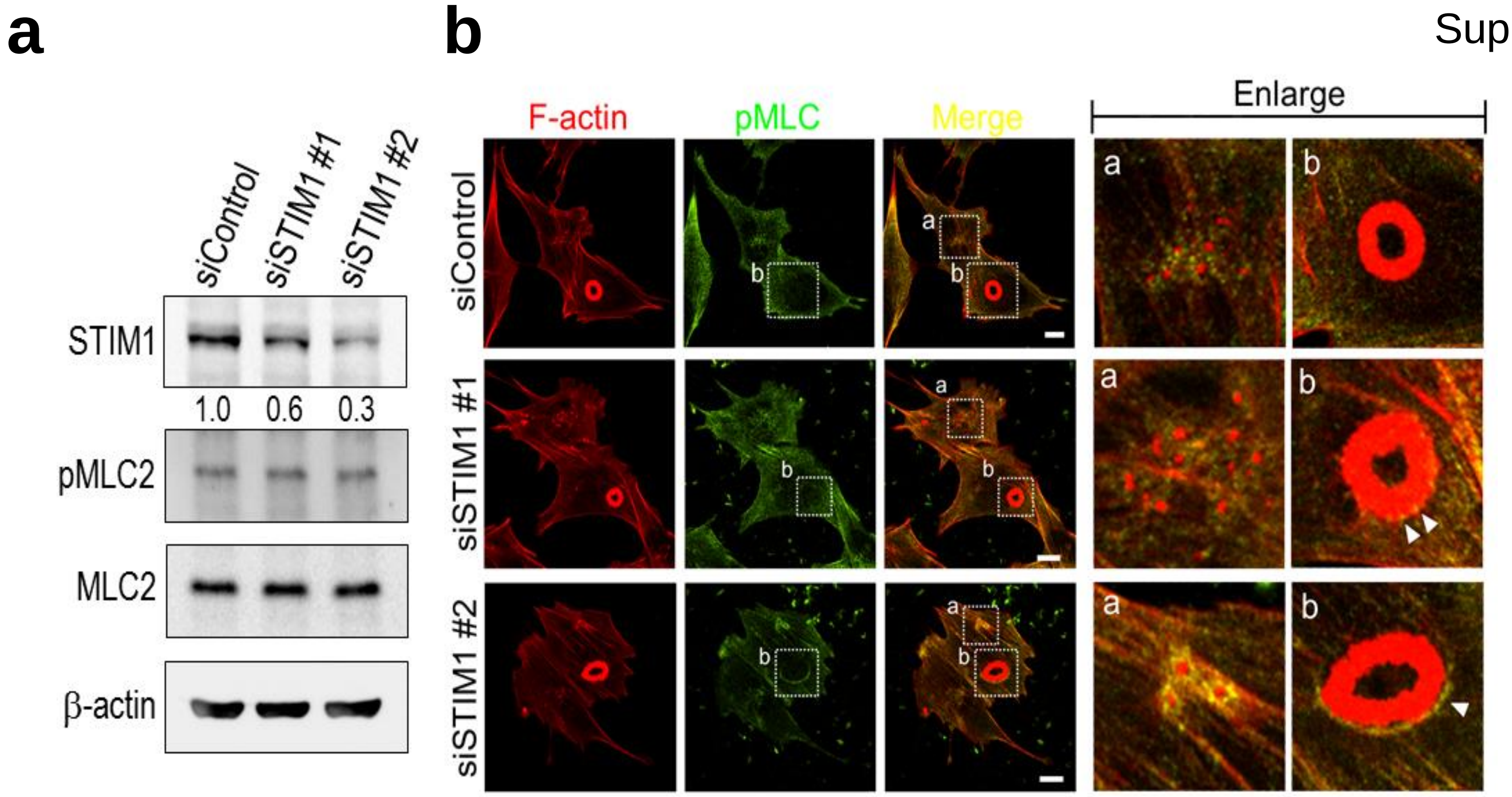

Supplementary Figure S3. Blockade of STIM1-mediated Ca^{2+} signaling changes distribution of actomysin in podosome rosettes. ***(a)*** The protein level of STIM1, phospho-myosin light chain (pMLC) and myosin light chain (MLC) in v-Src-transformed MEFs. Cropped blots have been presented. Full length blots are presented in Supplementary Fig. S13 ***(b)*** The representative confocal images showed the distribution between actomyosin and podosome rosettes. *Scale bar*, 10 μm. *Arrow*, the actomysin.

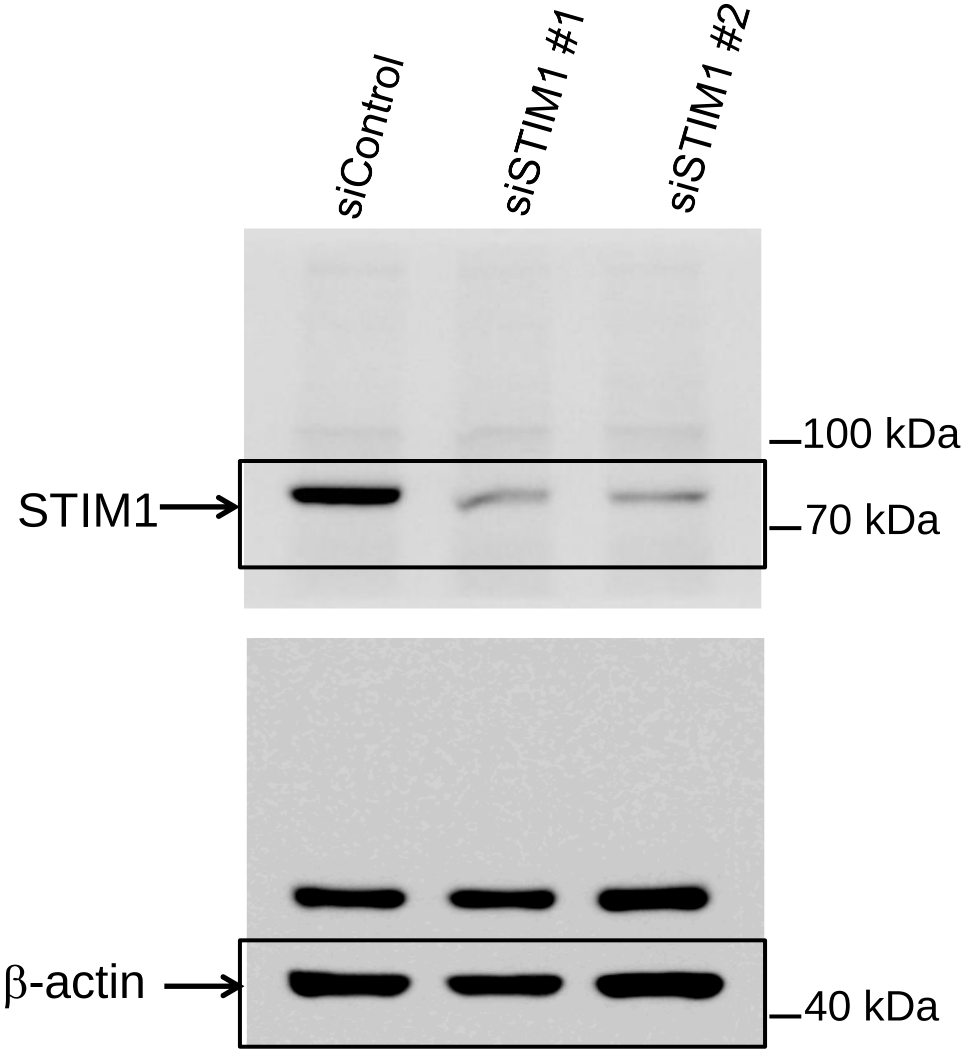

Supplementary Figure S4. Full length images of the cropped blots presented in main Figure 2a. Full length blots in Figure S4 showed that knockdown efficiency of siControl, siSTIM1 in breast cancer MDA-MB-231 cells. β-actin was used as a loading control for total protein.

Suppl. Fig 5

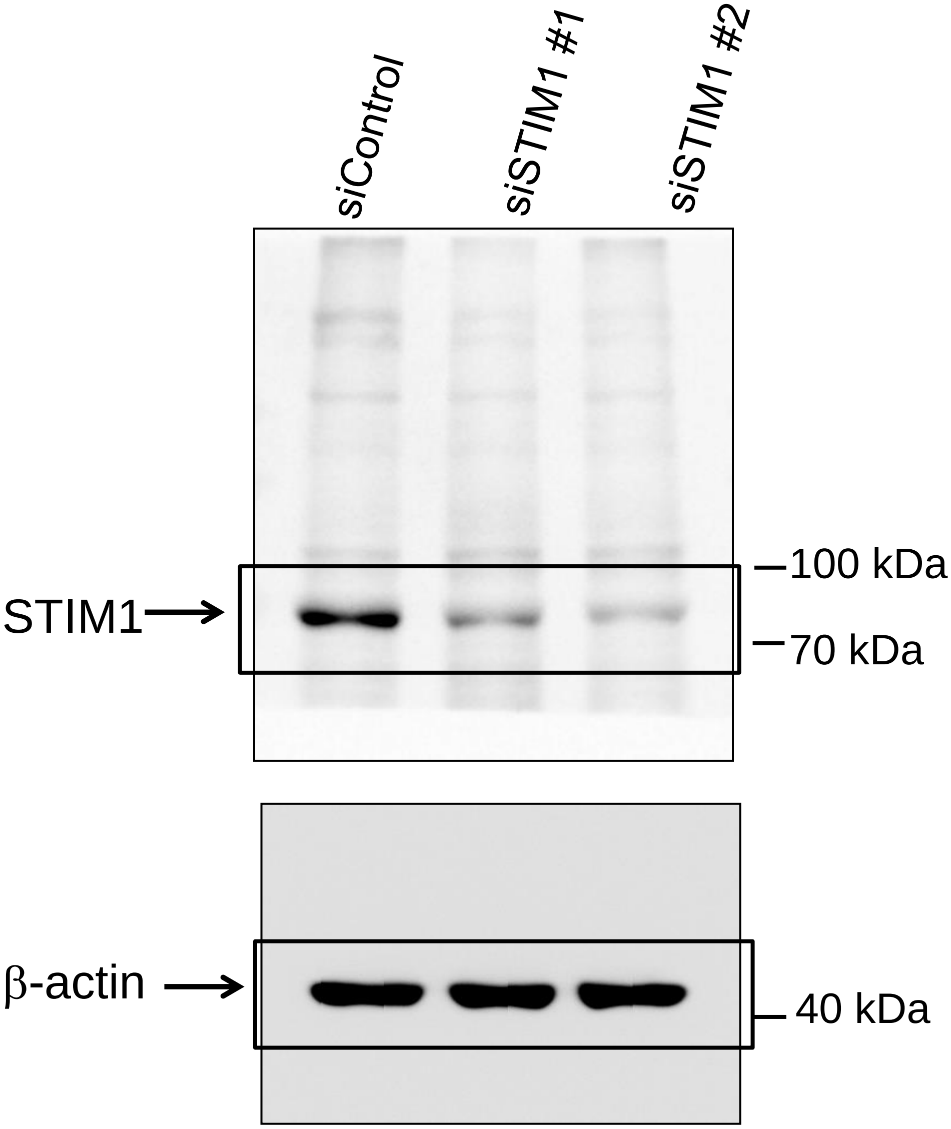

Supplementary Figure S5. Full length images of the cropped blots presented in main Figure 2d. Full length blots in Figure S5 showed that knockdown efficiency of siControl, siSTIM1 in osteosarcoma U2OS cells. β-actin was used as a loading control for total protein.

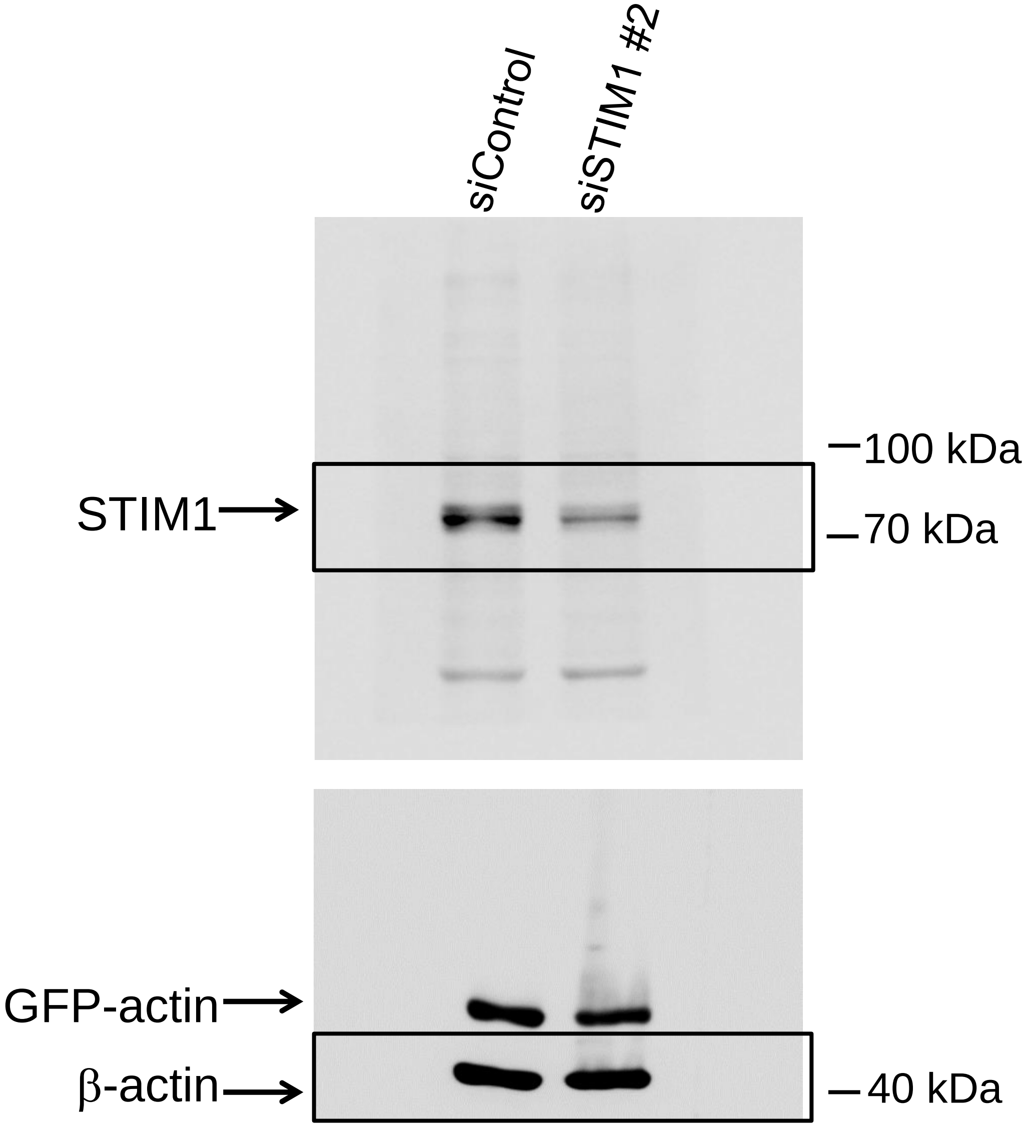

Supplementary Figure S6. Full length images of the cropped blots presented in main Figure 7a. Full length blots in Figure S6 showed that knockdown efficiency of siControl, siSTIM1 in v-Src-transformed MEFs. β-actin was used as a loading control for total protein.

Suppl. Fig 7

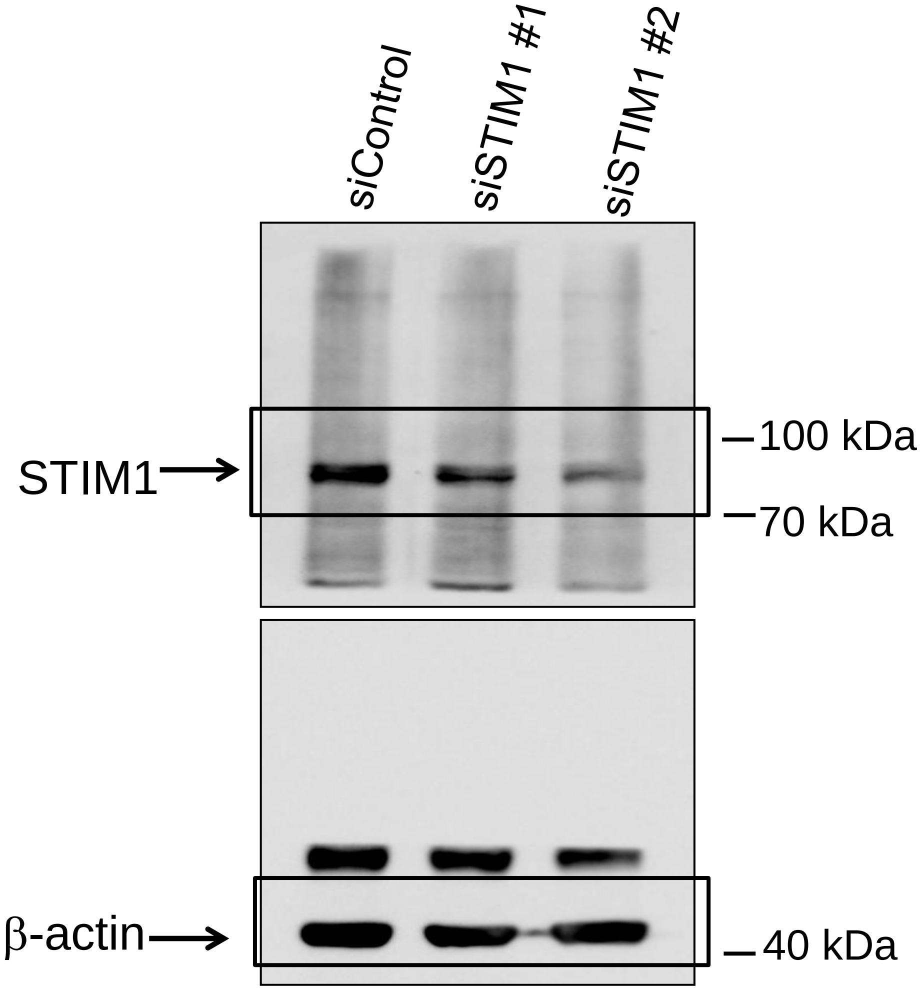

Supplementary Figure S7. Full length images of the cropped blots presented in main Figure 8a. Full length blots in Figure S7 showed that knockdown efficiency of siControl, siSTIM1 in v-Src-transformed MEFs. β-actin was used as a loading control for total protein.

Suppl. Fig 8

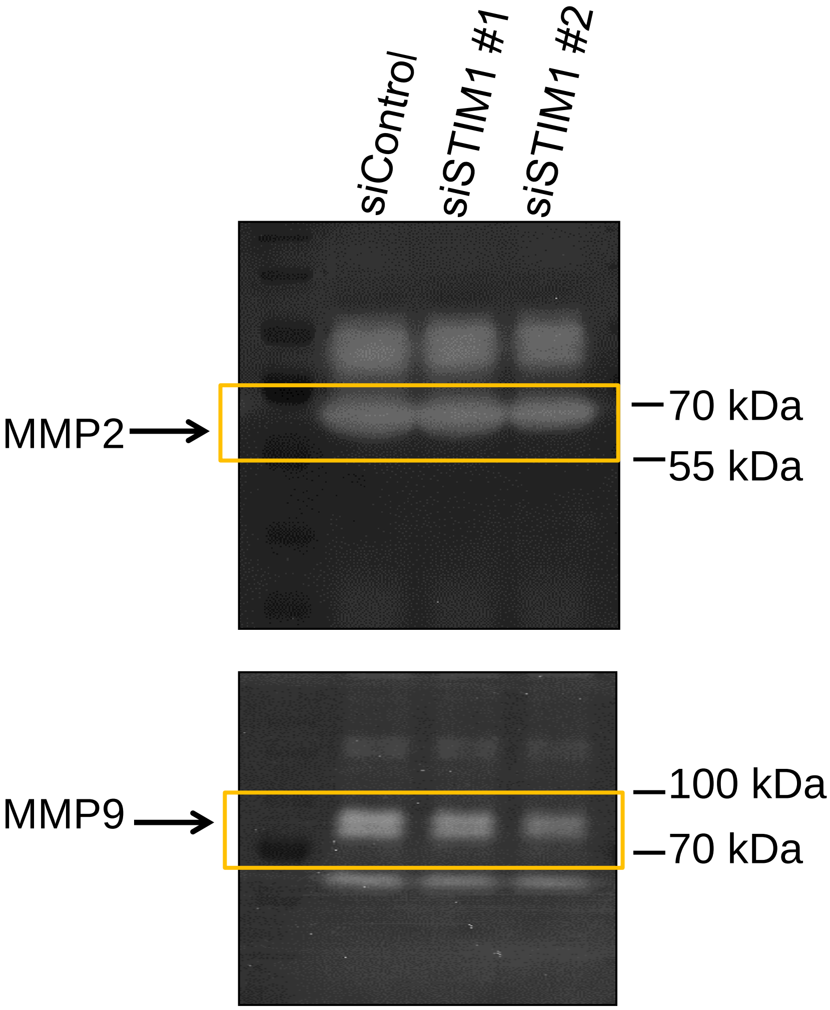

Supplementary Figure S8. Full length images of the cropped blots presented in main Figure 8b. Full length blots in Figure S8 showed representative zymograms showing activities of MMP2 and MMP9 in the conditioned medium. Equal volume (40 μL) of conditioned medium collected from 10-cm dish with equal cell number was loaded into each well.

Suppl. Fig 9

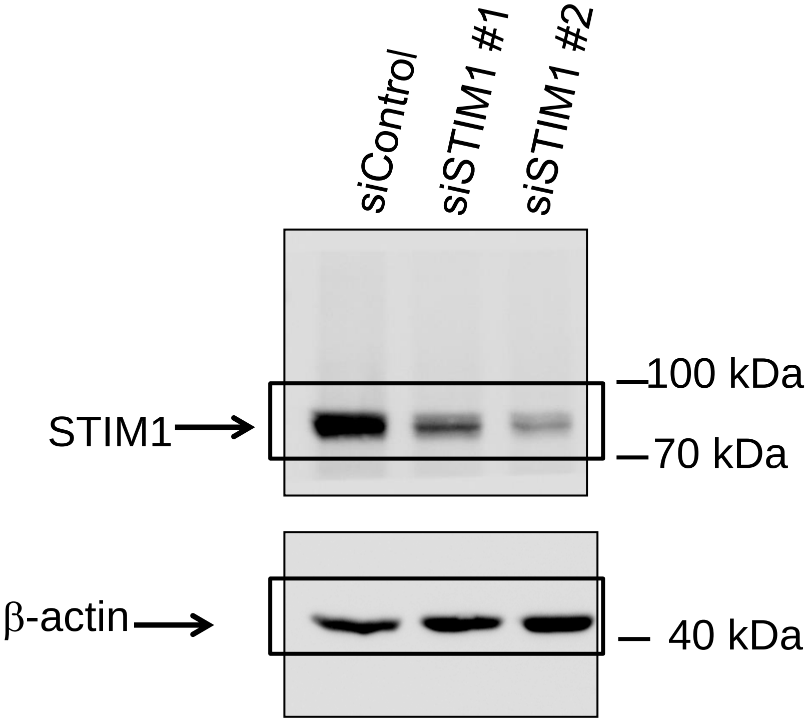

Supplementary Figure S9. Full length images of the cropped blots presented in main Figure S1a. Full length blots in Figure S9 showed knockdown efficiency of siControl, siSTIM1 in v-Src-transformed MEFs. β-actin was used as a loading control for total protein.

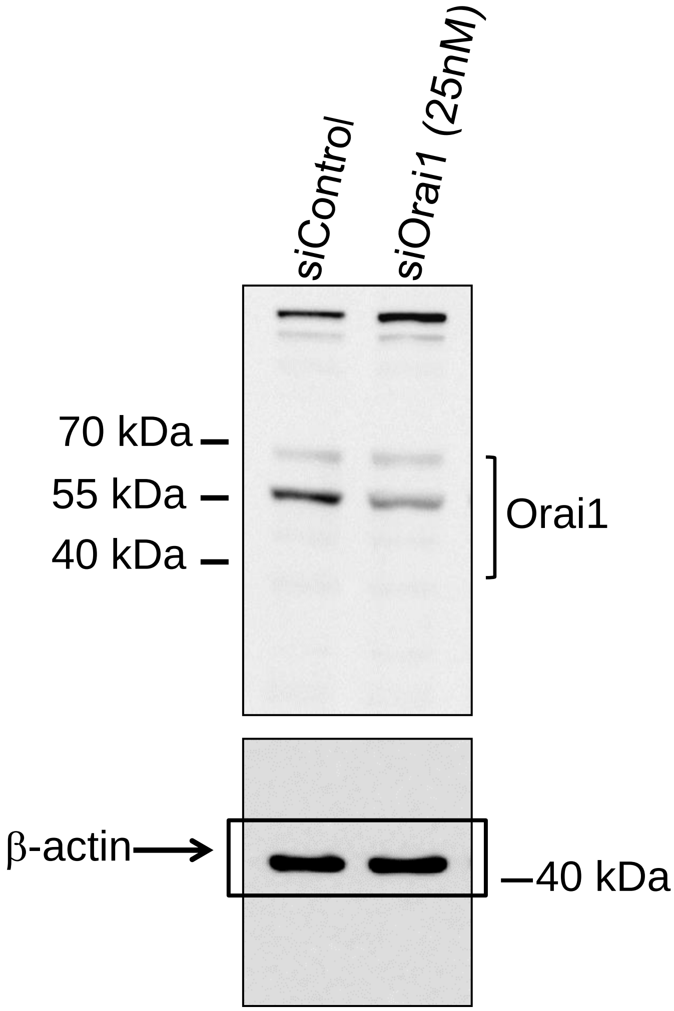

Supplementary Figure S10. Full length images of the cropped blots presented in main Figure S1b. Full length blots in Figure S10 showed knockdown efficiency of siControl, siOrai1 in v-Src-transformed MEFs. β-actin was used as a loading control for total protein.

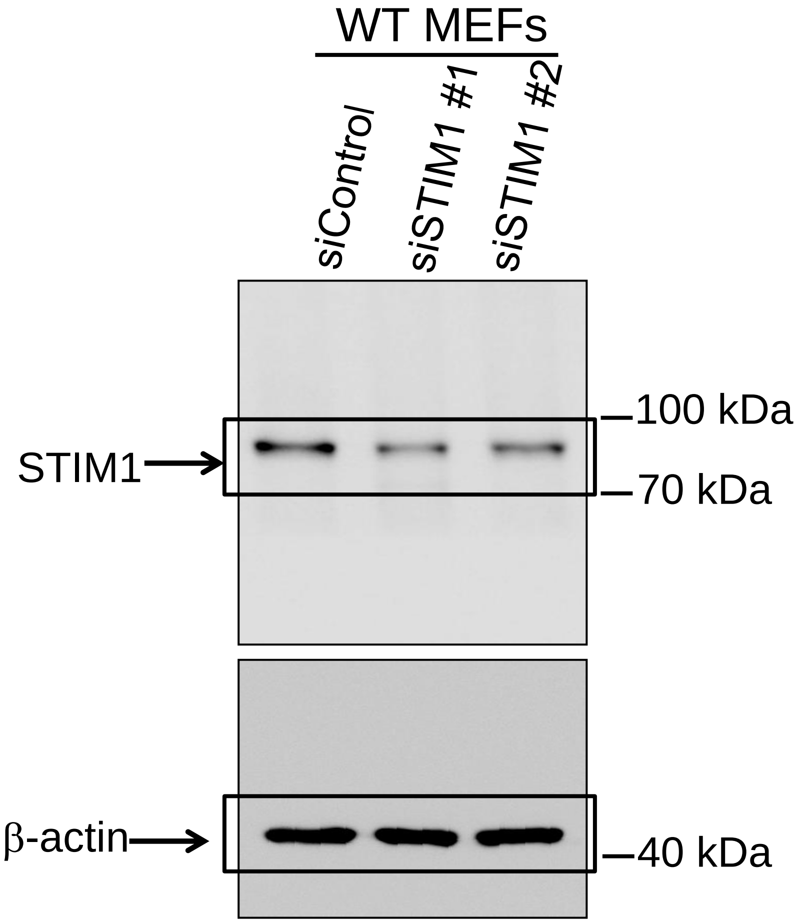

Supplementary Figure S11. Full length images of the cropped blots presented in main Figure S2a. Full length blots in Figure S11 showed knockdown efficiency of siControl, siSTIM1 in wild-type MEFs. β-actin was used as a loading control for total protein.

Suppl. Fig 12

B

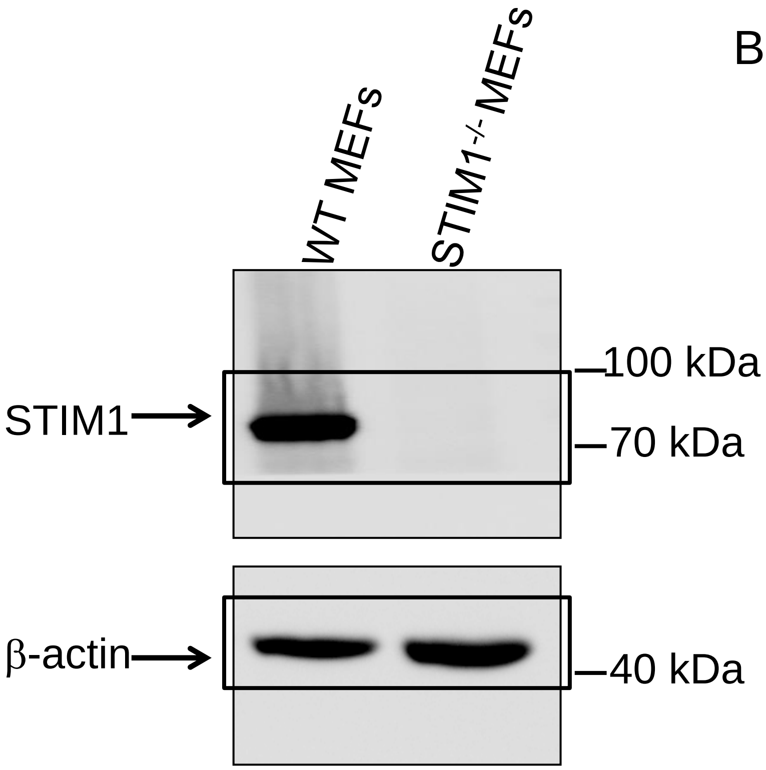

Supplementary Figure S12. Full length images of the cropped blots presented in main Figure S2b. Full length blots in Figure S12 showed Western blot analysis of expression pattern of STIM1 in MEF lacking STIM1 (STIM1$^{-/-}$ MEF).

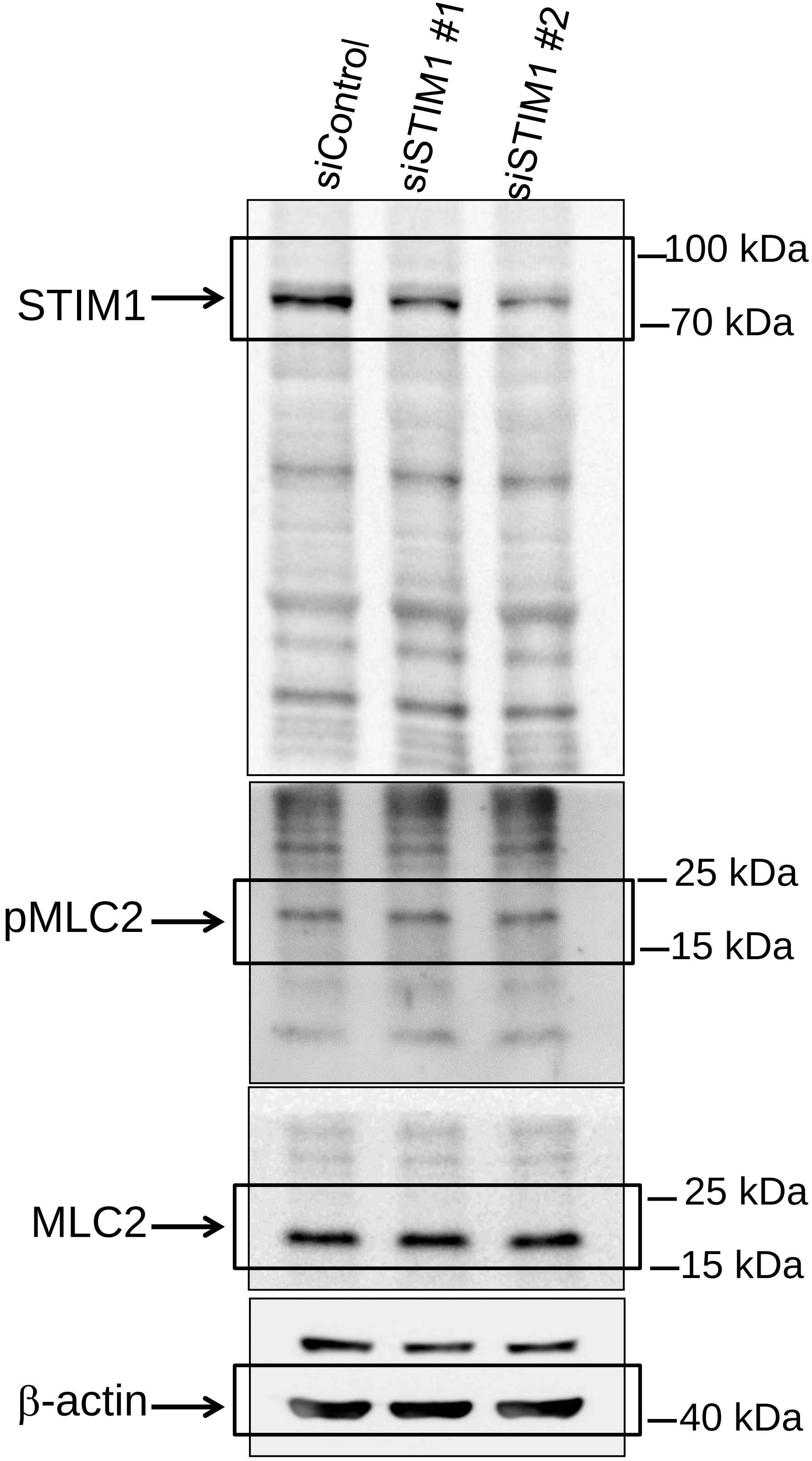

Supplementary Figure S13. Full length images of the cropped blots presented in main Figure S3a. Full length blots in Figure S13 showed Western blot analysis of expression pattern of STIM1, phospho-myosin light chain (pMLC) and myosin light chain (MLC) in v-Src-transformed MEFs.
